# Junctional Adhesion Molecule 3 Expression in the Mouse Airway Epithelium Is Linked to Multiciliated Cells

**DOI:** 10.3389/fcell.2021.622515

**Published:** 2021-07-28

**Authors:** Clara Maria Mateos-Quiros, Sergio Garrido-Jimenez, Guadalupe Álvarez-Hernán, Selene Diaz-Chamorro, Juan Francisco Barrera-Lopez, Javier Francisco-Morcillo, Angel Carlos Roman, Francisco Centeno, Jose Maria Carvajal-Gonzalez

**Affiliations:** ^1^Departamento de Bioquímica, Biología Molecular y Genética, Facultad de Ciencias, Universidad de Extremadura, Badajoz, Spain; ^2^Aìrea de Biologiìa Celular, Facultad de Ciencias, Universidad de Extremadura, Badajoz, Spain

**Keywords:** Jam3, tight junctions, airway epithelial cells, multiciliated cells, endosomes

## Abstract

Tight-junction (TJ) proteins are essential for establishing the barrier function between neighbor epithelial cells, but also for recognition of pathogens or cell migration. Establishing the expression pattern and localization of different TJ proteins will help to understand the development and physiology of the airway. Here we identify that the junctional adhesion molecule 3 (*Jam3*) expression is restricted to multiciliated cells (MCCs) in the airway epithelium. *In vitro*, Jam3 expression varies along airway basal stem cell (BSC) differentiation and upon DAPT treatment or IL6 exposure. However, Jam3 is not required for BSC differentiation to specific cell types. In addition, we found that MCC lacking Jam3 display normal cilia morphology and cilia beating frequency with a delay in BB assembly/positioning in MCCs during differentiation. Remarkably, Jam3 in MCC is mostly localized to subapical organelles, which are negative for the apical recycling endosome marker Rab11 and positive for EEA1. Our data show that Jam3 expression is connected to mature MCC in the airway epithelium and suggest a Jam3 role unrelated to its known barrier function.

## Introduction

Junctional adhesion molecule 3 (Jam3), together with Jam1 and Jam2, is known as a tight-junction (TJ) component that belongs to the junctional adhesion molecule family of proteins (Hartmann et al., 2020). In vertebrate cell cultures, Jam3 has been involved in epithelial barrier function; however, Jam3 knockout mice display additional phenotypes related with cell migration of immune cells and cilia and/or polarity in spermatids, airway epithelium, and brain ventricles (Kummer and Ebnet, 2018; Hartmann et al., 2020). Importantly, 40% of the lethality observed in Jam3-KO mice is due to pulmonary dysfunction, but how this phenotype is related to its cellular and subcellular localization related is completely unknown.

Since its discovery in 2004 (Cunningham et al., 2000), various *in vitro* studies have suggested that Jam3 has important functions in the assembly of TJs in endothelial and epithelial cells, but also in transendothelial and transepithelial migration of immune cells (Bazzoni, 2003; Ebnet, 2017; Hartmann et al., 2020). Indeed, Jam3 has been proposed to promote neutrophil migration *in vivo* and *in vitro* in endothelial cells (Chavakis et al., 2004; Woodfin et al., 2011). This Jam3-dependent neutrophil migration phenotype was also described *in vitro* with epithelial cells, where Jam3 is a component of desmosomes (Zen et al., 2004). A later study on Jam3 KO mice reported a pulmonary dysfunction where histological analysis revealed large infiltrates of neutrophils in the lungs in all moribund Jam3 KO mice (Imhof et al., 2007). Moreover, the blockade of Jam3 improved lung histology and reduced neutrophil contents in lungs of septic mice (Hirano et al., 2018). All this work supports a function of Jam3 in the lungs related to inflammatory response. On the other hand, under certain pathological conditions like cystic fibrosis (CF) or infection, TJs can be altered due to the inflammatory response (Coyne et al., 2002). The increased secretion of proinflammatory factors like tumor necrosis factor alpha (TNF-alpha) and interferon gamma (IFN-gamma)-increased secretion affects TJ structure and protein expression (Coyne et al., 2002). Interleukin 13 (IL-13), which drives asthma symptoms or IL-4 and IL-13 enhanced by IFN-gamma, induces changes in the TJ protein composition so that paracellular permeability becomes increased (Ahdieh et al., 2001; Schmidt et al., 2019).

As mentioned above, the initial studies *in vivo* using Jam3 KO mice showed that the deletion of Jam3 is to a large extent lethal, but the remaining male mice were infertile mice (Gliki et al., 2004). A closer look at the phenotype revealed that those Jam3 knockout mice had deficiencies in spermatid differentiation, when they change from round spermatids into spermatozoa, due to defect in the assembly of the polarity complex (Gliki et al., 2004). This could be considered as the first connection between Jam3 and cilium, taking into account that frequently mutations that generate motile cilia defects, like those found in primary ciliary dyskinesia (PCD), also have an effect on sperm tail formation. This is more evident if we look at the sperm tail and motile cilium basic structure. Both cellular components shared the same ultrastructural 9 + 2 microtubular arrangement (Sironen et al., 2020). More recently, Wyss et al. (2012) reported that Jam3 KO mice developed hydrocephalus, a phenotype that could also be related to cilia malfunctioning. In the brain, ependymal cell lining of cerebral ventricles covered their apical surface by cilia that beat in a coordinated fashion to facilitate circulation of the cerebrospinal fluid (CSF) (Tissir et al., 2010; Boutin et al., 2014; Carvajal-Gonzalez et al., 2016a). In the airway but also in other tissues, planar cell polarity signaling within cell–cell junctions properly orients cilia in multiciliated cells (MCCs) along the tissue (Carvajal-Gonzalez et al., 2016a). This function/connection between centriole positioning and PCP is a conserved function from flies to humans (Vladar et al., 2012, 2016; Carvajal-Gonzalez et al., 2016a,b; Garrido-Jimenez et al., 2019). In the literature, no connection has been described so far between Jam3 and PCP.

Here, we decided to characterize the molecular and cellular function in the airway epithelium of Jam3 and found that the expression of the junctional adhesion molecule, Jam3, is restricted to MCCs in the airway. In those cells, Jam3 localized mostly to apical sorting endosomes but did not contribute to cilia number, size, or morphology. However, Jam3 depletion affects basal body alignment and positioning at the apical side of MCC. On the other hand, prior to cell differentiation, Jam3 expression is upregulated in basal stem cells (BSCs) most likely to favor TJ formation. Overall, our findings place Jam 3 expression closely linked to MCC in the airway epithelium.

## Results

### Jam3 Expression *in vivo* Is Limited to Multiciliated Cells in the Mouse Airway Epithelium *in vivo*

**FIGURE 1:**
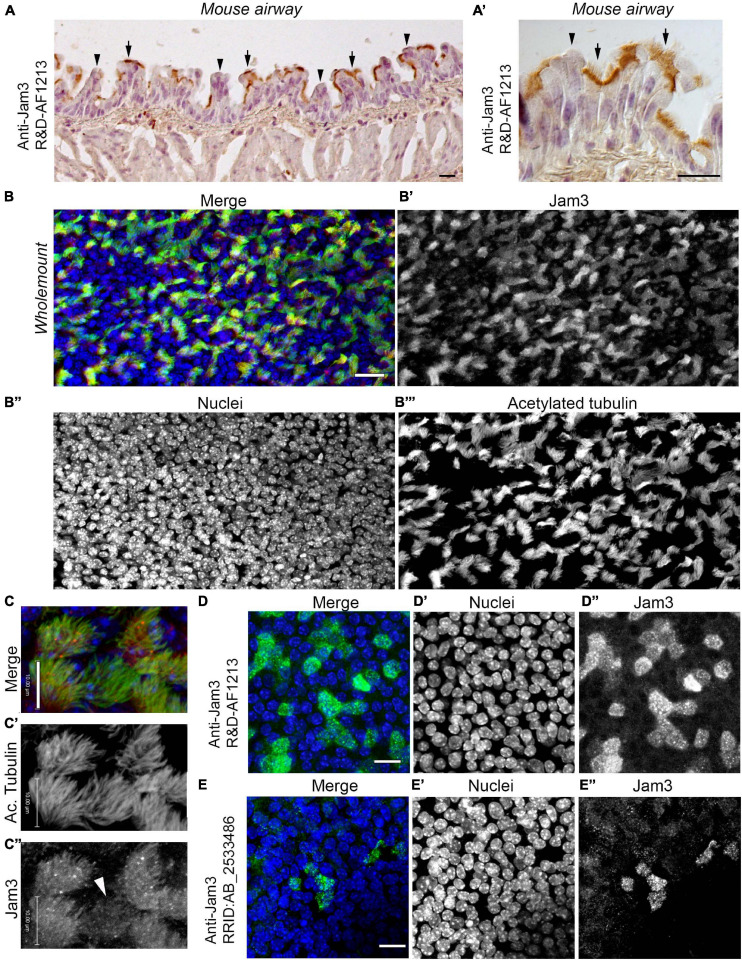
Junctional adhesion molecule 3 (Jam3) is expressed in multiciliated cells in the mouse airway epithelium. **(A)** Immunohistochemistry of Jam3 in the mouse airway epithelium. Black arrows point to multiciliated cells, while black arrowheads point toward non-ciliated cells. **(A’)** A higher magnification image for a Jam3 immunohistochemistry in the mouse airway epithelium. **(B’)** A magnification of an immunohistochemistry image of Jam3 in the mouse airway epithelium. **(B)** Immunofluorescence in mouse whole-mount trachea for Jam3 in red (gray in panel **B’**), acetylated tubulin in green (gray in panel **B’**), and DAPI in blue (gray in panel **B”**). **(C)** Confocal image with higher magnification for Jam3 localization in whole-mount tracheas, Jam3 in red (gray in panel **C’**), and acetylated tubulin in green (gray in panel **C”**). **(D,E)** Jam3 immunofluorescence in MTECs differentiated for 14 days *in vitro*, nuclei in blue (gray in panels **D’,E’**), and Jam3 in green (gray in panels **D’,E”**) using two different antibodies. Scale bar represents 10 μm in panel **(C)**, represents 10 μm, and represents 20 μm in panels **(D,E)**. White arrowhead point a MCC with low Jam3 expression levels.

The airway epithelium is formed mainly by three cell types, BSCs, secretory cells (SCs), and MCCs (Cardoso, 2001; Rawlins and Hogan, 2006). BSCs are responsible for tissue regeneration, and they are able to completely regenerate the epithelium (Rock et al., 2009; Yang et al., 2018). SCs, either club or goblet cells, produce mucus, essential to trapping pathogens and particles (Rawlins et al., 2009). Besides, MCCs play their role by constantly swapping the mucus toward the mouth, so that the airway tract remains cleared (Bustamante-Marin and Ostrowski, 2017). The Jam3 expression pattern in the airway is completely unknown, even though mice lacking the Jam3 gene die due to respiratory tract infections (Imhof et al., 2007). We started by performing an immunohistochemical analysis of Jam3 in the mouse airway. In mouse lung sections, we first observed that Jam3 localization is not homogeneous in the epithelium, but instead is restricted to some cells (Figures 1A,A’). A closer look at those cells revealed that Jam3 expression was presented in MCCs and not in secretory or basal neighbor cells (Figure 1A’). Likewise, a co-staining with Jam3 and acetylated tubulin, which accumulates in the cilium axoneme, in a whole-mount trachea showed that Jam3 was indeed co-localizing with MCCs labeled with acetylated tubulin (Figures 1B–B”’). We also noticed that in our whole-mount confocal images, Jam3 expression levels were heterogeneous among MCCs, where we can find a mix of cells with low or high Jam3 levels (Figures 1C–C”). Overall, we found that Jam3 expression in the airway epithelium is restricted to MCCs.

### Jam3 Expression *in vitro* Is Also Restricted to MCCs

To further characterize the localization and function of Jam3 in MCCs, we tested the localization pattern of Jam3 *in vitro*. We used differentiated mouse tracheal epithelial cell cultures (MTECs), which at least contain all three major cellular components, basal cells, SCs, and MCCs. Using two different antibodies (AF1213 and AB_2533486), we found a restricted endogenous expression of Jam3 in some cells (Figures 1D–D”,E–E”). To be completely sure that we were detecting Jam3, we cloned the cDNA of Jam3 from MTECs in frame with GFP to confirm that the Jam3 antibody was indeed detecting Jam3 in our immunofluorescence and also in Western blots (Supplementary Figure 1). A dog cell line [Madin-Darby Canine Kidney (MDCK) cells] and a human cell line (HEK293) were transfected with our Jam3-GFP construct (Supplementary Figure 1). We found that the Jam3 antibody AF1213 was capable of labeling only those MDCK cells expressing mouse Jam3-GFP and not neighbor cells (Supplementary Figure 1). In Western blot, the Jam3 antibody AF1213 and GFP antibody detected the expression of Jam3 only in those cells transfected with our Jam3-GFP (Supplementary Figure 1). This set of experiments confirmed that we were detecting mouse Jam3 with the antibody AF1213 and that a different antibody (AB_2533486) provided the same staining pattern.

**FIGURE 2:**
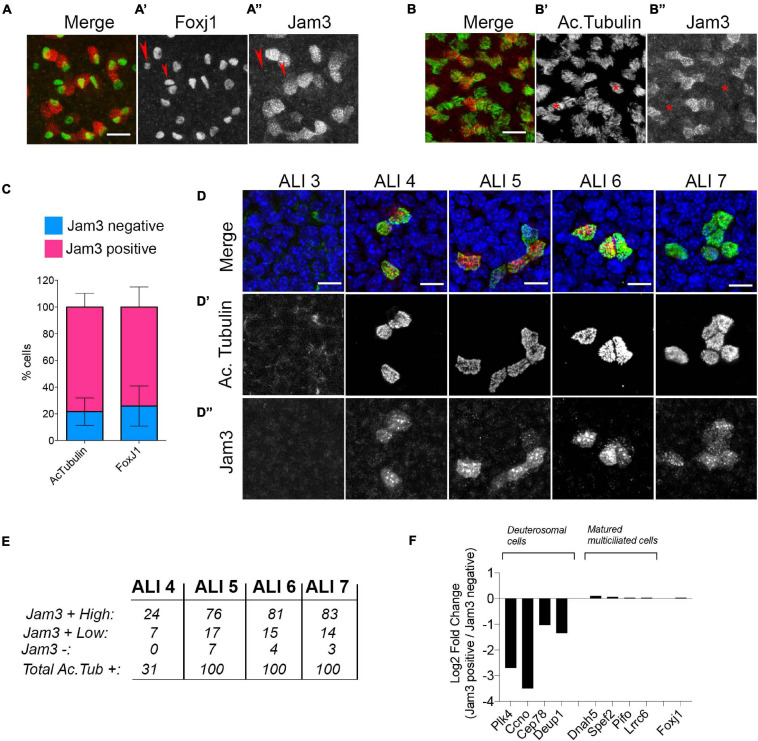
Junctional adhesion molecule 3 (Jam3) expression is restricted to a subset of multiciliated cells. **(A,B)** Representative images for Jam3 and Foxj1 **(A)** or acetylated tubulin **(B)** to evaluate the co-labeling of MCCs. Foxj1 in green (gray in panel **A’**), and Jam3 in red (gray in panel **A”**) Acetylated tubulin in green (gray in panel **B’**), and Jam3 in red (gray in panel **B”**) **(C)** Quantification of the number of cells that express Jam3 and acetylated tubulin or Jam3 and Foxj1. **(D)** Set of confocal images at different days of differentiation (from ALI 3 to ALI7) for Jam3 in red (gray in panel **D”**), acetylated tubulin in green (gray in panel **D’**), and nuclei in blue. **(E)** Quantification of acetylated tubulin-positive cells with high levels of Jam3, low levels of Jam3, or negative for Jam3 from ALI4 to ALI7. No acetylated tubulin or Jam3-positive cells were detected in ALI3. **(F)** Relative gene expression levels for deuterosomal cells and mature ciliated cell markers in Jam3-positive or negative cells (all of them are Foxj1-positive cells). Scale bar in panels **(A,B)** represents 20 and 10 μm in panel **(D)**. Red arrow pointed Foxj1 positive cells which are Jam3 negative. Red starts marked Ac-tubulin positive cells which are Jam3 negative.

Next, we performed two different double immunofluorescences with MCC markers, Jam3 and Foxj1 (a transcription factor required for MCC differentiation) and Jam3 and acetylated tubulin (Figures 2A–A”,B–B”). Upon imaging and quantification, we found that above 75% of cells that were positive for Foxj1 or acetylated tubulin were also clearly positive for Jam3 (Figure 2C). We determined that Jam3 expression in the airway epithelium is restricted to MCCs *in vivo* and *in vitro*; however, there are some cells *in vitro* which are negative for Jam3 that we were not able to detect *in vivo*.

To test whether this Jam3 expression was related to a differentiation status of MCCs, we co-stained MTECs with Jam3 and acetylated tubulin along the differentiation process when MCCs start to appear in this culture system (from ALI3 to ALI7) (Figures 2D–D”). We found that acetylated tubulin started to accumulate and assemble cilia from ALI4 and that it is not detected in ALI3 (Figure 2D’). In those conditions we found that Jam3 also accumulates in some cells (only around 30 cells in a 6.5-mm filter with thousands of cells) from ALI4 and that the number of cells co-labeled with both proteins, Jam3 and acetylated tubulin, increased along the differentiation process (Figure 2D and quantified in Figure 2E). In our immunofluorescence images, we could find acetylated tubulin-positive cells with high levels of Jam3, which were the majority of the cells in all conditions from ALI4 to ALI 7 (Figure 2E). Yet, we could also find acetylated tubulin cells with lower levels of Jam3 and very few cells with acetylated tubulin cells and without Jam3 staining (Figure 2E).

To further characterize the connection between Jam3 expression and MCC differentiation status, we evaluated the expression of genes that distinguish between deuterosomal cells or mature MCCs (Bukowy-Bieryłło, 2021) using published single-cell expression data sets (Plasschaert et al., 2018). Our analyses revealed that those MCCs that do not express Jam3 had higher expression levels of deuterosomal cell markers, while no difference between Jam3-positive or -negative cells are found for mature MCC markers or Foxj1 (Figure 2F).

Based on these results, we concluded that most of MCCs at steady state are Jam3-positive cells and that Jam3 expression in MCCs is linked to mature MCCs.

### Jam3 Subcellular Localization in MCC Is Abundant in Apical Sorting Endosomes

**FIGURE 3:**
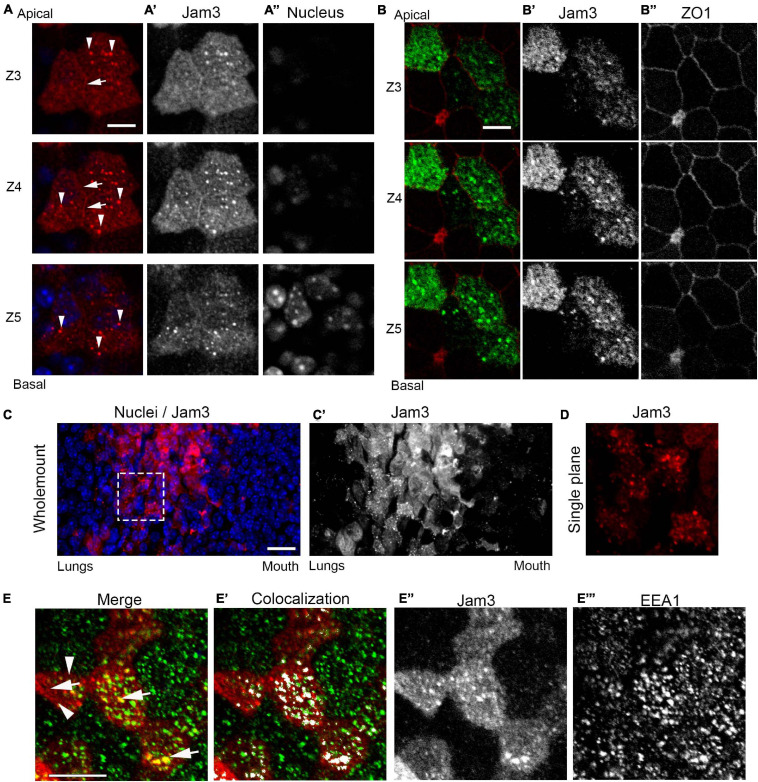
Junctional adhesion molecule 3 (Jam3) localizes at cell–cell contacts and in apical sorting endosomes. **(A)** Confocal Z-planes from apical to more basal ones (Z3 to Z5) of Jam3 in MCCs. White arrows point to Jam3 localization at cell contacts, and white arrowheads denoted Jam3 localization in apically located endosomes. Jam3 in red (gray in panel A’), and nucleus in blue (gray in panel A”). **(B)** Serial confocal Z-planes (Z3 to Z5) of Jam3 co-localization with ZO1 in MCCs. Jam3 in green (gray in panel B’), and ZO1 in red (gray in panel B”). **(C)** Immunofluorescence in mouse whole-mount trachea for Jam3 in red (gray in panel **C’**) and nuclei in blue. **(D)** A zoom for a region of interest from panel **(A)** showing Jam3 localization in a group of cells. **(E)** A single confocal plane image for Jam3 in red (gray in panel **E”**) and EEA1 in green (gray in panel **E”’**) co-localization analyses (white in panel **E’**). White arrows point to Jam3 and EEA1 co-localization in endosomes, and white arrowheads denoted Jam3 endosomes which are EEA negative. Note that not all EEA1-positive endosomes are positive for Jam3. The step size between Z planes is 1 μm. Scale bar in panels (**A,B,D**) represents 10 μm. The dotted white box represents the ROI depicted in panel **(D)**.

As observed in Figure 1B’, Jam3 localization in MCCs is at the apical portion of the cell and not detected in the distal part of the cilia axonemes or along the basolateral side of these epithelial cells. This subcellular distribution was confirmed by immunofluorescence and confocal imaging of Jam3 in MTECs (Figures 3A–A”,B–B”). By looking at the different confocal plane images, we were able to detect low levels of Jam3 at the cell–cell contacts (white arrows in Figure 3A and Supplementary Figure 2), but we also detected Jam3 expression above those junctions and in very apically located endosomes at the TJ plane stained with ZO1 (Figure 3B). Looking back in our whole-mount staining confocal images, we could also find Jam3 located in endosomes in the most apical planes (Figures 3C–C’,D).

To describe the endosomal nature of those organelles where Jam3 localizes in MCCs, we performed co-staining of Jam3 with an apical sorting endosome marker, EEA1, and an apical recycling endosomal marker, Rab11. We found that many of the Jam3-containing endosomes were also apical-sorting endosomes co-labeled with EEA1 (Mander’s coefficients, M1 = 0.28 and M2 = 0.34; displaced images: M1 (five-pixel shift) = 0.12 and M2 (five-pixel shift) = 0.13; M1 vs. M1 (five-pixel shift) *p* = 0.03 and M2 vs. M2 (five-pixel shift) *p* = 0.01; see section “Materials and Methods”) (Figures 3E–E”” and Supplementary Figure 2) and co-localization with Rab11 was almost not found (Supplementary Figure 2). Recently, liquid-like organelles have been related to cilia development (Lee et al., 2020); we tested whether Jam3 appeared in those organelles where Daap1 or Dnai1 was concentrated and found no co-staining in MCCs (Supplementary Figure 2). To further locate Jam3 at the membrane, we did a co-staining of Jam3 with Vangl1, a planar cell polarity-related protein localized in adherent junctions. By looking at different images at the most apical planes of MTECs, we found that junctional Jam3 is located more apically than Vangl1 (Supplementary Figure 3), although this Jam3 localization at the membrane is not always easy to observe.

Overall, Jam3 localized in MCCs mostly at apical-sorting endosomes at the level of the TJs and in some cells is detected at the cell–cell contacts above Vangl1 junctions.

### Cilia Morphology and Function Are Not Altered in the Absence of Jam3

**FIGURE 4:**
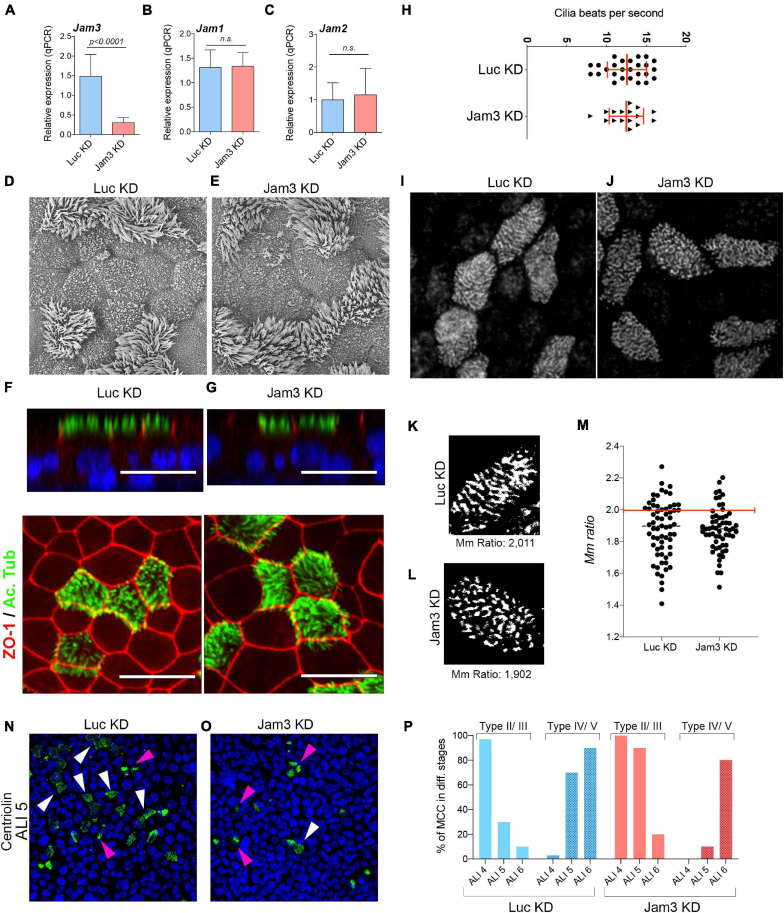
Downregulation of Jam3 does not alter cilia structure and function. **(A–C)** Mean mRNA expression levels of Jam3 **(A)**, Jam1 **(B)**, and Jam2 **(C)** assessed in differentiated MTECs infected with control viruses (Luc KD) or Jam3-shRNAs (Jam3 KD), *n* = 3. **(D,E)** Scanning electron micrographs of Jam3-KD and control (Luc KD) MTECs differentiated for 14 days. **(F,G)** Lateral and top views of confocal images for acetylated tubulin (in green), ZO-1 (in red), and nucleus (in blue) in Jam3-KD **(G,G’)** and Luc KD **(F,F’)** MTECs. **(H)** Cilia beating frequency quantification as number of beats per second in cells treated with Luc KD and Jam3 KD MTECs. **(I,J)** Basal body staining in control **(I)** and Jam3 KD cells **(J)**. **(K,L)** Black and white image obtained to calculate the Mm ratio in a control cell **(K)** and a Jam3 KD cell **(L)**. **(M)** Mm ratio quantification for individual cells. *n* > 60 in Control and Jam3 KD conditions. **(N,O)** Centriolin staining in control (Luc KD) and Jam3-KD cells in ALI 5. **(P)** Quantification of MCCs in different stages of differentiation in control and Jam3 KD cells. Type II/III are those cells with Centriolin staining in aggregates (pink arrowheads) while Type IV/V are those cells with Centriolin staining dispersed at the apical membrane (white arrowheads). Scale bar in panels **(F,G)** represent 20 μm. *p*-values in all conditions were obtained using *t*-test.

Mouse tracheal epithelial cell cultures have been extensively used to study the biology of the airway, including cell differentiation, cilia functioning, or protein secretion, among many other features. To assess Jam3 function in the airway epithelium, we developed a combination of shRNAs capable of decreasing the expression of Jam3 in MTECs to study the function of Jam3 in MCCs. Briefly, during cell expansion and prior to MTEC differentiation, we infected and selected MTECs with Jam3 shRNAs to produce the desired Jam3 knockdown (Jam3-KD). For Jam3-KD, we used a combination of two shRNAs to bolster the knockdown efficiency, as single shRNA provided less knockdown capability. As a control, we infected MTECs with a shRNA against luciferase (Luc-KD). Jam3 downregulation was confirmed at the RNA level by quantitative PCR in MTECs differentiated in the air liquid interface for 14 days (ALI 14) (Figure 4A) and at the protein level by immunofluorescence (Supplementary Figure 4). Notice that in those Jam3-KD conditions, we could not detect Jam3-positive cells that were easily found in control Luc-KD conditions (Supplementary Figure 4). We assessed the expression of Jam1 and Jam2 in Jam3-KD conditions compared to Luc-KD and found that Jam3 downregulation does not affect the expression of the other two junctional adhesion molecules (Figures 4B,C).

Once our Jam3-KD was settled and based on the cellular and subcellular localization of Jam3 in MCCs, we first decided to look at cilia morphology using scanning electron microscopy (SEM). As shown in Figure 4D,E, no morphological ciliary defects were detected in SEM micrographs in Jam3-KD cells when compared to Luc-KD cells (Figures 4D,E). We also found a similar ciliary size and cilia number at the apical membrane of MTECs (Figures 4D,E). Next, we performed an immunofluorescence against acetylated tubulin and found no differences between control and Jam3-KD conditions (Figures 4F,F’,G,G’). These experiments supported that cilia structure, morphology, or number was not affected by Jam3 depletion, but is cilia functioning affected?

To assess a role in cilia function, we developed a new assay including a graphic user interface (GUI) to measure cilia beating. In short, we added magnetic beads at the apical chamber of differentiated MTECs. After a few minutes, these beads dropped on top of cells and remained attached to their apical membrane (by a mechanism that we ignore). Due to its color and size, those magnetic particles can be easily visualized and recorded with a high-speed camera (Supplementary Figure 5 and Supplementary Movie 1). We tested this new method exposing MTECs to well-known blockers of cilia beating. As shown in Supplementary Figure 4, cold PBS with calcium and magnesium could slow down cilia beating from 15 to 2 bps. Cilia beating could also be completely blocked with nickel chloride (Supplementary Figure 5). Once the system was set, we assessed cilia beating in control and Jam3-KD conditions and found the same beating capacity when compared to control conditions (Figure 4H).

Next, we analyzed the basal body distribution pattern in MCCs. To this end, we performed basal body staining in WT and Jam3 KD MCCs. Then, we used a similar approach as in Herawati et al. (2016) which discriminated between non-aligned and aligned basal bodies along MCC differentiation. A custom-made GUI quantified the alignment of basal bodies in individual MCCs using eccentricity and the major/minor axis ratio (Mm ratio) of these bodies (see materials and methods section for more detailed information). In both measures, we would find longer values in the case of elongated (and more aligned) ROIs. We found that in WT cells, BBs were distributed in well-aligned long rows in above 40% of the cells; however, in Jam3 KD cells very few cells contained well-aligned long rows of BBs (Figures 4I–M). Instead, most of Jam3 KD cells contained short arrays of BBs. Noticeably, when we looked at microtubules or actin staining, we did not find major differences between WT and Jam3 KD cells (Supplementary Figure 5), so this BB distribution pattern defect in Jam3 KD does not depend on the cytoskeleton. However, the analyses of BB distribution together with the differential expression of deuterosomal cell markers suggested that Jam3 could be affecting BB migration or attachment to the apical membrane. To test this hypothesis, we perform Centriolin staining along the early stages of monolayer differentiation (from ALI 4 to ALI 6) in WT and Jam3 KD cells. It has been reported that the Centriolin staining pattern is a good marker to categorize MCCs during differentiation. MCCs can be found in stage I with a pair of centrioles, stage II/III with many centrioles aggregated and migrating, and stage IV/V with many centrioles homogeneously distributed at the apical membrane (Usami et al., 2021). We found that Jam3 KD cells had a significant delay in MCC maturation since only 10% of MCCs are in stage IV/V in ALI 5 compared to 70% in control cells (Figures 4N–P). This difference is less dramatic in ALI 4 and ALI 6 (Figure 4P).

In summary, we found that Jam3 downregulation did not seem to affect cilia morphology or function but it mildly affected BB organization and assembly in MCCs.

### Jam3 Expression Downregulation in the Airway Epithelium Does Not Dramatically Affect Epithelium Integrity

As presented in the introduction, the role of junctional adhesion molecules, Jam1, Jam2, and Jam3, in epithelial cells is mostly related to epithelial barrier function. Based on this knowledge, we decided to explore the function of Jam3 in the mouse airway epithelium monolayer, even though its expression is restricted to MCCs mostly in endosomes.

**FIGURE 5:**
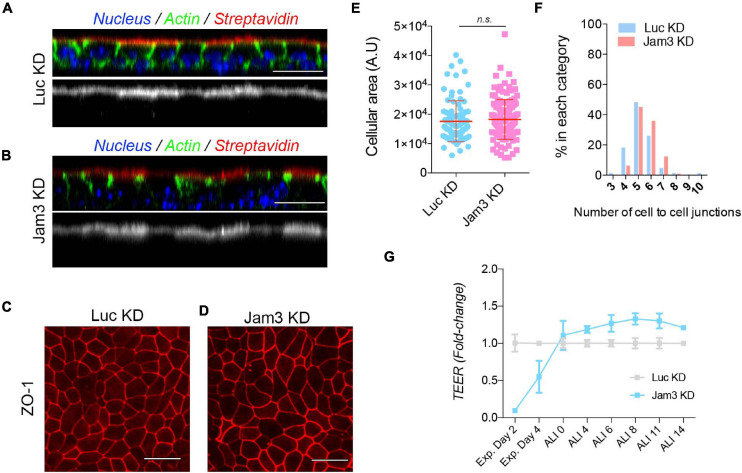
Downregulation of Jam3 expression does not affect epithelial integrity but delays airway epithelial monolayer TEER during expansion. **(A)** Confocal images corresponding to orthogonal views in MTECs control **(A)** and Jam3 KD cells **(B)**. Streptavidin Alexa 555 (in red) was only observed at the apical membrane. Phalloidin (in green) was used to label the cortical actin in both the apical and basolateral surfaces and Dapi (in blue) for nucleus. **(C,D)** Confocal images to evaluate ZO-1 recruitment to the junction in Luc KD **(C)** and Jam3 KD **(D)** cells. **(E,F)** Cell size **(E)** and cell–cell contact number **(F)** evaluation in confocal images of ZO-1 in Luc KD and Jam3 KD cells. **(G)** Transepithelial resistance (TEER) measure was used to test tight-junction permeability during the differentiation process of MTECs in Jam3-KD compared to Luc-KD cells. Scale bar in panels **(A–D)** represents 20 μm. *p*-values in all conditions were obtained using *t*-test.

To measure epithelial integrity, we first tested how leaky the epithelium was in Jam3-KD vs. Luc-KD conditions. We performed a differential extracellular biotinylation assay (see Materials and Methods for experimental details) in MTECs and found that in both Jam3-KD and Luc-KD, the pool of biotinylated proteins at the plasma membrane revealed by Streptavidin-555 was restricted to the apical membrane (Figures 5A,B). This experiment demonstrates that even in the absence of Jam3 the airway epithelia formed a continuous monolayer that could not be trespassed by a small molecule like biotin.

To further characterize the epithelial monolayer, we performed a ZO1 staining, to check for cell size, cellular packing, and ZO-1 recruitment to the junctions in the absence of Jam3. We did not find a dramatic change in cell size, cell shape, or ZO-1 staining in Jam3-KD cells when compared to Luc-KD cells (Figures 5C–F). Based on our data, we concluded that the epithelial integrity was not affected in absence of Jam3.

Finally, we tested the TJ permeability to ions in the monolayer by measuring the transepithelial resistance (TEER) prior polarization and along the differentiation process of MTECs in Jam3-KD compared to control Luc-KD cells. We observed that Jam3-KD and Luc-KD displayed similar TEER values during the differentiation process in the air liquid interface, from ALI0 to ALI14 (Figure 5G), although Jam3-KD cells showed a slightly higher TEER when compared to control cells. Additionally, we found that in Jam3-KD cells the airway epithelial monolayer without differentiation, still during expansion, had a delay in reaching the maximum TEER. Measurements of TEER in day 2 and day 4 during expansion were significantly lower in Jam3-KD cells when compared to Luc-KD cells (Figure 5G). However, staining of adhesion molecules like E-cadherin or ZO1 did not show major differences in pre-ALI cultures in control vs. Jam3-KD cells (Supplementary Figure 6).

Altogether, we concluded that Jam3 epithelial integrity in differentiated airway epithelial cells is not dramatically compromised.

### Jam3 Expression Is Downregulated Along Differentiation in MTECs but Does Not Affect BSC Differentiation

**FIGURE 6:**
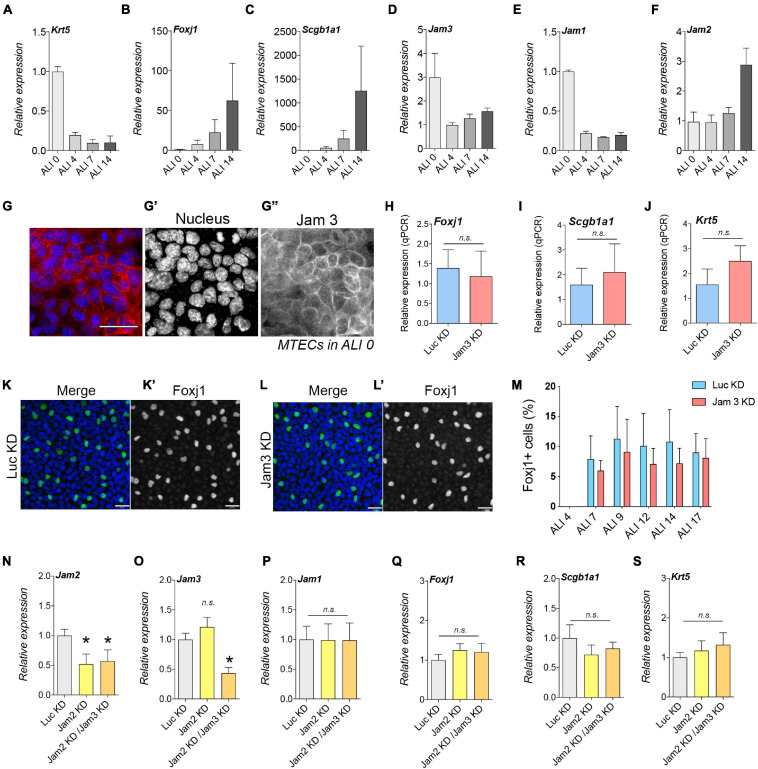
Junctional adhesion molecule 3 (Jam 3) expression varies along the differentiation process but does not affect cell differentiation. **(A–C)** Quantification of mRNA expression of Krt5 (basal marker, in panel **A**), FoxJ1 (multiciliated cells marker in panel **B**), and Scgb1a1 (secretory cells marker in panel **C**) expression in ALI 0, ALI 4, ALI 7, and ALI14. **(D–F)** Quantification of mRNA expression of Jam1 **(E)**, Jam2 **(F)**, and Jam3 **(D)** expression in ALI 0, ALI 4, ALI 7, and ALI14. Mean relative to control cells and standard deviation as error bars were plotted for each lineage marker, *n* = 4. **(G)** Jam3 immunofluorescence in BSCs during expansion *in vitro*, nucleus in blue (gray in panel **G’**) and Jam3 in red (gray in panel **G”**). **(H–J)** mRNA expression levels of Krt5 **(J)**, FoxJ1 **(H)**, and Scgb1a1 **(I)** in Luc KD **(C)** and Jam3 KD **(D)** MTECs. Mean relative to control cells and standard deviation as error bars were plotted for each lineage marker, *n* = 4. **(K,L)** Confocal images for Foxj1 (green in panels **K,L** and gray in panels **K’,L’**) immunofluorescence in Luc KD **(K)** or Jam3 KD **(L)** cells in ALI14. **(M)** Relative quantification of Foxj1-positive cells along the differentiation process in Luc KD and Jam3 KD cells from ALI 4 to ALI 17. **(N–S)** Mean mRNA expression levels of Jam3 **(O)**, Jam1 **(P)**, Jam2 **(N)**, Krt5 **(D)**, Foxj1 **(E)**, and Scgb1a1 **(F)** assessed in differentiated MTECs infected with control viruses (Luc KD), Jam2-shRNAs (Jam2 KD), and double knockdown (Jam2 and Jam3), *n* = 4. Scale bar in panels **(G,K,L)** represents 20 μm. *p*-values in all conditions were obtained using the *t*-test. *p < 0.05.

So far, we have found that Jam3 is required for proper junction formation before cell differentiation, but how is that possible if Jam3 is not expressed in BSC *in vivo*? We decided to measure Jam3 expression during the differentiation process by quantitative PCR. Control markers for different cell types were used to assess the differentiation process (Figures 6A–C). In ALI0, we detected high expression levels for *Krt5* but no detectable levels of *Scgb1a1* or *Foxj1*. As *Krt5* started to decrease its expression, *Foxj1* and *Scgb1a1* expression progressively appeared from ALI4 to ALI14 (Figures 6A–C). Unexpectedly, *Jam3* expression was high in ALI0 and dramatically decreased in ALI4 (Figure 6D). *Jam1* displayed a similar expression profile when compared to *Jam3* (Figure 6E), and the opposite was observed for *Jam2*, with higher levels at day 14 of differentiation (Figure 6F).

We decided to confirm this Jam3 expression pattern by immunofluorescence and found that prior to cell differentiation, Jam3 expression is clearly detectable in BSCs. As shown in Figure 6G–G”, Jam3 was homogeneously expressed in all cells in the monolayer. Moreover, Jam3 localization was mostly at the plasma membrane at the cell–cell contacts. This result was in complete agreement with our qPCR data and TEER measurements, where Jam3 expression was high in ALI0 (Figure 6D).

Since Jam3 expression suffered this severe change in expression during differentiation and it is restricted to MCCs, could Jam3 affect BSC differentiation to MCCs? To test this hypothesis, we differentiated MTECs in Jam3-KD and Luc-KD and checked the expression of different cell-type markers. We found that downregulation of Jam3 did not affect *Foxj1*, *Scgb1a1*, or *Krt5* expression when compared to Luc-KD conditions (Figures 6H–J). In addition, immunofluorescence analyses revealed that the number of Foxj1-positive cells along the differentiation process had no significant differences in MCC number from ALI7 to ALI17 in control vs. Jam3-KD conditions (Figure 6K,K’,L,L’,M). Finally, since *Jam2* expression severely changed along the differentiation process and could compensate our Jam3 depletion, we knocked down Jam2 alone and Jam2 in combination with Jam3 (double Jam2-KD/Jam3-KD) (Figure 6N–P). As already found in Jam3, Jam2 KD or Jam2-KD/Jam3-KD did not affect the expression of *Jam1* (Figure 6P). Similar to our Jam3 data during differentiation, single Jam2-KD or double Jam2-KD/Jam3-KD did not affect BSC differentiation as no changes were found in the expression of *Krt5*, *Foxj1*, or *Scgb1a1* (Figures 6Q–S).

Therefore, neither Jam3 nor Jam2 is related to the initial differentiation of BCs to MCCs even though their expression varies along the differentiation process.

### Jam3 Expression Is Enhanced in the Presence of IL6, but Jam3 Localization Remains in Endosomes and Not at the Plasma Membrane

**FIGURE 7:**
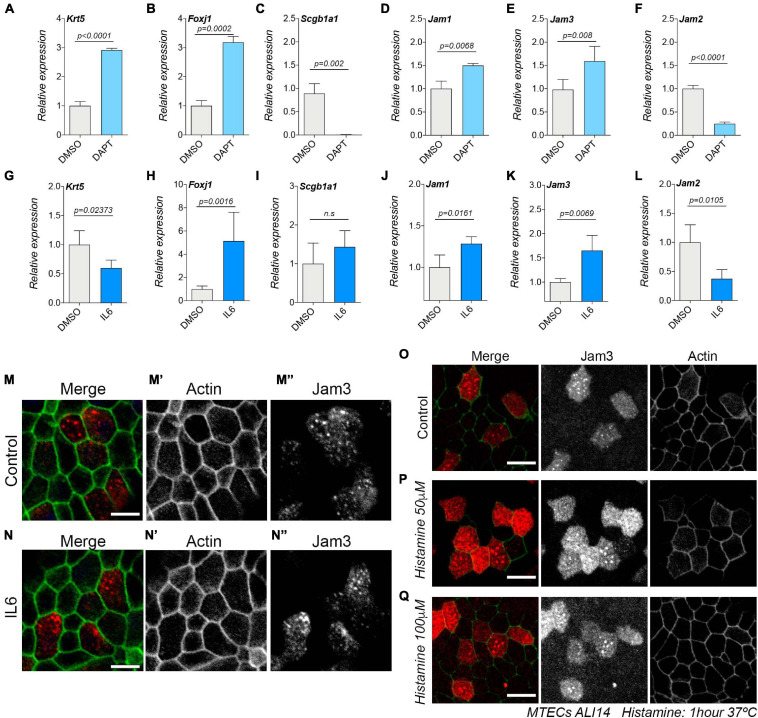
Junctional adhesion molecule 3 (Jam 3) expression is enhanced in MTECs treated with IL6 during differentiation. **(A–F)** mRNA expression levels of Krt5 for basal cells **(A)**, Foxj1 for MCCs **(B)**, and Scgb1a1 for club cells **(C)**, Jam1 **(D)**, Jam3 **(E)**, and Jam2 **(F)** in MTECs treated with DMSO or DAPT for 14 days. **(G–L)** mRNA expression levels of Krt5 for basal cells **(G)**, Foxj1 for MCCs **(H)**, and Scgb1a1 for club cells **(H)**, Jam1 **(I)**, Jam3 **(K)**, and Jam2 **(L)** in MTECs treated with PBS or IL6 for 14 days. Mean expression values relative to DMSO-treated cells and standard deviation as error bars were plotted for each lineage marker, *n* = 4. **(M–N)** Jam3 localization in MTECs treated for 14 days with PBS **(M)** or IL6 **(N)**. The cell membrane was labeled using actin in green (gray in panels **M’,N’**) and Jam3 in red (gray in panels **M”,N”**). **(O–Q)** Jam3 localization in MTECs treated for 1 h with PBS **(O)** or two concentrations of histamine **(P,Q)**. Scale bar in panels **(M–Q)** represents 20 μm. *p*-values in all conditions were obtained using the *t*-test.

We have found that Jam3 is restricted to MCCs *in vivo* and *in vitro* and that Jam3 expression does not modulate the amount of MCCs. However, we wonder if Jam3 expression and localization could be modulated by or in MCCs. We first treated MTECs with DAPT, a well-established method to inhibit Notch signaling (Pardo-Saganta et al., 2015). Notch inhibition treatment provoked a shift during the differentiation process in MTECs, promoting MCC and BSC differentiations and abolishing SCs. Upon DAPT treatment of MTECs for 14 days, we confirmed an increase in BSC and MCC markers, *Krt5* and *Foxj1*, respectively (Figures 7A,B), while expression of the SC maker *Scgb1a1* was not detected (Figure 7C). Under those experimental conditions, we observed an increase in *Jam3* and *Jam1* expression and a decrease in *Jam2* expression (Figures 7D–F). These data support that Jam3 expression is linked to MCCs and that Jam2 expression could be more related to SC.

As mentioned in the introduction, TJ component expression can be also modulated by the inflammatory response. Interleukin-6 (IL6) together with IFNγ is a proinflammatory cytokine related to neutrophil trafficking during the inflammatory response, and Jam3 has been related to neutrophil migration (McLoughlin et al., 2003). In addition, IL6-driven signaling *via* STAT3 limits the inflammatory recruitment of neutrophils and Jam3 knockout mice showed an abnormal accumulation of neutrophils in the lungs (Imhof et al., 2007; Fielding et al., 2008). Lastly, IL6 can modulate the amount of MCCs in the mouse and human airway epithelium (Tadokoro et al., 2014). Based on this literature, we decided to test whether the expression and localization of Jam3 could be modulated by IL6 by looking at its expression levels upon IL6 treatment. As expected, IL6 increased the amount of MCCs in MTECs in ALI14, which correlates with an increased expression of *Foxj1* (Figures 7G–I). Under those experimental conditions, we also found that *Jam3* expression was upregulated (Figures 7J–L). However, we did not find a different subcellular pattern of localization for Jam3 in MCCs when compared to control treated cells (Figures 7M–M”,N–N”). We concluded that Jam3 expression, but not its subcellular localization, can be regulated by IL 6 because more MCCs developed in the airway epithelium.

Finally, it has been shown that Jam3 accumulated in endosomes can be mobilized from endosomal compartments toward the plasma membrane in endothelial cells upon short exposure to histamine or VEGF (Orlova et al., 2006; Kostelnik et al., 2019). Therefore, we decided to test whether endosomal Jam3 in MCCs could be mobilized by histamine. We found that the localization pattern in MCCs did not change after a 1-h exposure to either 50 μM histamine or 100 μM histamine or PBS (as a control) (Figures 7O–Q). Hence, unlike in endothelial cells, histamine cannot mobilize the pool of Jam3 accumulated in endosomes toward the plasma membrane.

## Discussion

There have been initial studies on regional/spatial TJ protein expression along the respiratory tract. For example, claudin expression varies along the epithelium, and the proximal section expresses Cldn- 1, 3, 4, 5, 7, 10, and 18-1 and the distal section Cldn- 3, 4, 7, 8, 15, and 18-1 (Günzel and Yu, 2013). How all those localization patterns contribute to the physiology of the respiratory tract requires detailed analyses for each of those proteins. Jam3 knockout mice die mostly due to respiratory dysfunction, and yet the localization and cellular function in the airway epithelium remained unknown. Here we established Jam3 as a protein that expressed only MCCs in the mouse airway epithelium with no effect on BSC differentiation, epithelial integrity, or major cilia defects. We described that Jam3 protein acts early during BSC monolayer establishment to allow proper TJ formation but later on Jam3 function is restricted to mature MCCs where Jam3 is mainly located at the apical membrane and apical-sorting endosomes.

Junctional adhesion molecule 3 is known as a basolateral protein in epithelial cells, yet in this study we located Jam3 in an apical-related localization in apical-sorting endosomes. Previously, Jam3 was also located in other subcellular places like in germ/sertoli cell contacts where Jam3 is in the junctional plaques (Gliki et al., 2004; Cartier-Michaud et al., 2017). In these junctional plaques, Jam3 is involved in acrosome and cell polarity of the germ cells (Gliki et al., 2004; Pellegrini et al., 2011; Cartier-Michaud et al., 2017). Our unexpected apical localization in MCCs in the airway was also previously reported in the kidney proximal tubule, where the absence of a specific clathrin adaptor, AP1B, resulted in the apical localization in those epithelial cells (Schreiner et al., 2010). In PT epithelial cells, the role of Jam3 in this apical membrane remains unknown. It is also true that it is not the sole TJ protein “misplaced” at the apical membrane in an epithelial cell. We have previously identified that CAR was also localized to the apical membrane in an AP1B-dependent mechanism. But again, why some cells have CAR at their apical membrane is unknown (Diaz et al., 2009; Carvajal-Gonzalez et al., 2012; Gravotta et al., 2012). In this study, we found that an important pool of Jam3 was located intracellularly in apical sorting endosomes and not much at the basolateral membrane or TJs. One possibility is that Jam3 located in endosomes regulates the transport of certain cargo proteins that need to be delivered to the apical plasma membrane or even the cilia. Another possible function might be that Jam3 regulates the association of different proteins to apical-sorting endosomes like GTPases like RhoD or RhoB that can regulate the cytoskeleton (Fernandez-Borja et al., 2005; Nehru et al., 2013). This last scenario seems unlikely since we do not detect actin or tubulin defects in Jam3 KD cells at steady state. Further work will be required to answer how and why Jam3 reached this apical sorting endosome, but also how Jam3 affects BBs assembly/distribution operating from endosomes.

Our data using Jam3-KD cells did not show a massive cilium-related phenotype. We did not find a major cilia morphology, number, or beating defects. However, we found a more subtle defect in basal body distribution pattern in the absence of Jam3, which also correlates with a delay in BB assembly/positioning in MCCs. A proper basal body distribution at the apical membrane of this MCCs is responsible for making cilia to beat in the same direction (Carvajal-Gonzalez et al., 2016a; Adler and Wallingford, 2017; Roman et al., 2019). In addition, we did not find a planar polarized distribution of Jam3 or co-localization with a PCP core component like Vangl1. In summary, at this point we believe that Jam3 in mature MCCs might be required for proper basal bodies distribution in individual cells without an effect in the planar coordination along the epithelium.

## Materials and Methods

### Mouse Tracheal Epithelial Cell (MTECs) Primary Cell Preparation

Mouse tracheal epithelial cell cultures were obtained from adults wild-type C57BL/6J mice. All animal studies have been performed in accordance with the National and European legislation (Spanish Royal Decree RD53/2013 and EU Directive 86/609/CEE as modified by 2003/65/CE, respectively) and in accordance with the Institute of Laboratory Animal Resources (ILAR) for the protection of animals used for research. Experimental protocols were approved by the Bioethics Committee for Animal Experimentation of the University of Extremadura (Registry July 7, 2017). Tracheas were dissected from the larynx to the bronchial main and collected in cold 1× Ham-F12 (Gibco, Grand Island, NY, United States) with penicillin and streptomycin 1% (P/S, Gibco). Then, connective, fatty, and vascular tissues were removed in cold Ham-F12 P/S. After that, clean tracheas were cut longitudinally and incubated in Ham-F12 P/S containing 1.5 mg/ml pronase (Roche Molecular Biochemicals, Penzberg, Germany) for 16 h at 4°C. Fetal serum bovine (FBS) (Gibco) was added to a final concentration of 10%. Supernatant was transferred to a new tube, and Ham-F12 with 10% FBS was added to the tracheas. Contents of the tubes were mixed and centrifuged at 500 × *g* for 5 min. Pellets containing cells were resuspended in 5 ml of Ham-F12 P/S with 0.5 mg/ml pancreatic DNAse I (Sigma, St. Louis, MO, United States), incubated for 10 min, and centrifuged at 500 × *g* for 5 min. Finally, cells were resuspended in PneumaCult-Ex Plus complete medium (StemCell, Vancouver, BC, Canada) and preselected in Primaria tissue culture plates (Corning, Tewksbury, MA, United States) for 4 h in 5% CO2 at 37°C to remove fibroblasts. Cells were seeded in 60-mm plates previously treated with 50 μg/ml type I rat tail collagen (Gibco) in 0.02 N acetic acid.

### Cell Culture, Differentiation, and Treatments

Mouse tracheal epithelial cell cultures were expanded in PneumaCult-Ex Plus medium at 37°C in 5% CO_2_ until 70–80% confluence. Then, cells were incubated with 0.02% EDTA in PBS for 20 min and Accutase (Gibco) for 10 min at room temperature and counted. For differentiation, 9 × 10^4^ cells/cm^2^ were seeded in polyester porous membranes (Transwell 0.4-μm pores, Corning). The upper and lower chambers were filled with PneumaCult-Ex Plus medium, which was changed every 2 days. Once confluence was reached (4–6 days), the medium was removed from the upper chamber and changed in the lower chamber to ALI medium (Air-Liquid Interface Medium, StemCell). The ALI medium was replaced every 2 days until the end of the differentiation for 14 days (ALI 14).

For treatment, DAPT (Tocris, Bristol, United Kingdom) 50 μM in DMSO was added to the medium during differentiation of MTEC cultures starting in ALI 0. Dimethyl sulfoxide (DMSO) was used as control.

For histamine (#10745842, Acros Organics, Fair Lawn, NJ, United States) treatment, histamine was dissolved in PBS and added to the media at two different final concentrations (50 and 100 μM) to MTECs in ALI14 for 1 h at 37°C. PBS was used as control.

For recombinant IL6 mouse (#PMC0064, Gibco) treatment, MTECs were seeded in 6.5-mm Transwells and IL6 treatment with 10 ng/ml went from ALI1 to ALI14 (media was replaced every 2 days). PBS was used as control.

Madin-Darby Canine Kidney cells were expanded in Dulbecco’s modified Eagle’s medium (DMEM) (Gibco) at 37°C in 5% CO_2_ until 70%–80% confluence. Then, MDCK were incubated in PBS for 15 min and Trypsin-EDTA (0.25%) phenol red (Gibco) for 2 min at room temperature and counted. Twenty-five thousand cells were seeded in a 12-well plate with DMEM. The next day, cells were transfected with Jam3-PIG (puromycin-IRES-GFP) construction using Lipofectamine 2000 Transfection Reagent (Invitrogen) with Opti-MEM (Gibco) according to the manufacturer’s instructions. After 4 h, the medium was removed and changed to DMEM. After 2 days, cells were selected with puromycin 3 μg/ml throughout the experiment. When cells reached 70–80% confluence, they were seeded in 60 mm and finally were plated for experiments.

### Lentivirus Production and Infection

Short hairpin RNAs (shRNAs) against Jam3 and Jam2 were cloned in the pLKO.1 vector (see Supplementary Table 1 for sequences), which drives the expression of shRNAs from the U6 human promoter and contains a puromycin-IRES-mCherry selection cassette. Purified DNAs were transfected with the packaging vectors psPAX2 and PMD2.G into HEK-293T cells by using polyethylenimine (PEI) (Sigma). Viruses were collected 48 and 72 h post-transfection and concentrated with Amicon Ultra-15 (Merck) by centrifugation at 3500 × *g* for 30 min at 4°C up to an approximate concentration of 2 × 10^5^ infectious virus particles/ml. We added a volume of 50 μl of concentrated virus in 2 ml of medium in the presence of 8 μg/ml polybrene (Sigma). Infected MTECs were selected with puromycin 3 μM during 48 h. Finally, cells were expanded and plated for experiments.

For Jam3 overexpression, RNA was obtained from MTECs ALI 14 and retrotranscribed using SuperScript^TM^ III One-Step RT-PCR System with Platinum^TM^ Taq (Invitrogen, Carlsbad, CA, United States). Jam3 was amplified by PCR using the following oligonucleotides: 5′CCACAACCATGGCGCTGAGCCGG-3′, 5′CCATGGTTGTGGTCCAGATAACAAAGGACG-3′. Mouse Jam3 was cloned into a pLKO.1-containing puromycin-IRES-GFP vector using the BstXI (New England Biolabs, Ipswich, MA, United States) enzyme. Jam3 was cloned in frame with the GFP of this expression cassette. Chemically competent bacteria (One Shot Stbl3 competent *Escherichia coli*) (Invitrogen) were transformed and plasmid DNA was mini-prepped from two colonies, and the presence of insert was assessed by digestion with BstXI and by DNA sequencing.

### RNA Extraction, cDNA Synthesis, and qPCR

RNA was isolated from MTECs using Illustra RNAspin Mini Kit (GE Healthcare, Chicago, IL, United States). After elution, 200–400 ng RNA was reverse transcribed with cDNA reverse transcription Kit (Applied Biosystems, Foster City, CA, United States) according to the manufacturer’s instructions. Gene expression was analyzed by qPCR using PowerUp SYBR Green PCR Master Mix (Applied Biosystems) in QuantStudio 3 (Thermo Fisher) with specific primers (Supplementary Table 1). PCR reaction was set at 50°C for 2 min, 95°C for 10 min, and 50 cycles of 95°C for 15 s and 60°C for 1 min. Melt curve analysis was used to confirm the specificity of the reaction.

### Western Blot (WB) Analysis

HEK-293T cells transfected with a mCherry Control (sh Luciferase) and Jam3-GFP were lysed in ice-cold lysis buffer containing 50 mM Tris–HCl (pH 7.5), 1 mM EGTA, 1 mM EDTA, 1 mM sodium orthovanadate, 5 mM sodium pyrophosphate, 10 mM sodium fluoride, 0.27 M sucrose, 0.1 mM phenylmethylsulfonyl fluoride, 0.1% (v/v) 2-mercaptoethanol, 1% (v/v) Triton X-100, and complete protease inhibitor cocktail (Roche). The protein concentration was determined by Bio-Rad protein assay, and 20 μg protein was analyzed by SDS-PAGE electrophoresis and blotted onto nitrocellulose membranes (Bio-Rad Laboratories). The membranes were blocked with 5% dry milk Tris-buffered saline containing 0.05% Tween-20 during 1 h and were incubated with primary antibodies diluted in blocking solution overnight at 4°C. Antibodies used were anti-GFP (Roche, Basel, Switzerland, #11814460001, 1:500), anti-Jam3 (R&D Systems, Minneapolis, MN, United States, #AF1213, 1:500), and anti-vinculin (Sigma #V4505, 1:2000). The membranes were washed five times with TBS-Tween and incubated with horseradish peroxidase (HRP)-conjugated secondary antibodies (anti-goat-HRP, Cell Signaling (1:1000); anti-mouse-HRP, Cell Signaling #7076 (1:1000)) diluted in blocking solution for 1 h at room temperature. Finally, membranes were washed with TBS-Tween and proteins were detected using a chemiluminescence detection system (SuperSignal West Dura, Thermo Scientific) and iBright CL1000.

### Immunofluorescence

Whole-mount tracheas, MTECs in air liquid interface, or MTECs before reaching confluency were fixed in 4% PFA (PolyScience, Warrington, PA, United States) for 10 min at room temperature, permeabilized in PBS-Triton 0.1% for 15 min, and blocked in PBS-Triton with 2% bovine serum albumin (BSA) (Roche) for 1 h. Samples were incubated with primary antibodies anti-Jam3 (R&D Systems, #AF1213, 1:150), anti-Jam3 (Thermo Fisher, AB_2533486, 1:100), E-cadherin (BD Biosciences, #610182, 1:100), anti-ZO1 (1:100), Vangl1 (Sigma, HPA025235, 1:100), anti-FoxJ1 (Invitrogen, #14-9965-82, 1:200), AcTub (1;100), anti-Rab11a (Cell Signaling, Danvers, MA, United States, #2413S, 1:50) or anti-EEA1 (Cell Signaling, #3288S, 1:100), anti-Dnai1 (Thermo Fisher, PA554526, 1:100), anti-Daap1 (Sigma-Millipore, Burlington, MA, United States, HPA049468, 1:100), anti-centriolin (Santa Cruz, Santa Cruz, CA, United States, SE-365521, 1:100), and anti-alpha tubulin (Thermo Fisher, 32-2500, 1:100), diluted in PBS-Triton-2% BSA in a wet chamber overnight at 4°C. After that, samples were washed five times in PBS-Triton and incubated with fluorescent secondary antibodies [Alexa-Fluor-594 anti-Goat (Invitrogen, #A11058, 1:500), Alexa-Fluor 594 anti-Rat (Invitrogen, #A21209, 1:500), Alexa-Fluor 594 Phalloidin (Invitrogen, #A12381, 1:500), Alexa-Fluor 488 anti-Rabbit (Invitrogen, #A21206, 1:500), Alexa-Fluor 488 anti-Goat (Invitrogen, #A32814, 1:500), or Alexa-Fluor 488 anti-Mouse (Invitrogen, #A11001, 1:500)] diluted in PBS-Triton-2% BSA for 1 h at room temperature. DAPI 0.5 μg/ml (Thermo Scientific, #62248, 1:2,000) was used to label nuclei. Finally, samples were washed five times in PBS-Triton again and mounted in Vectashield (Vector Labs, Burlingame, CA, United States). Images were obtained using an Olympus FV 1000 confocal microscope and were processed using ImageJ (Fiji) and Adobe Photoshop CC 2019.

For cell size and packing analyses, images from MTECs ALI14 stained with ZO-1 were obtained by measuring the cell area in pixels and counting the number of sides of the cells to know the number of cell–cell junctions respectively using ImageJ (Fiji) and GraphPad Prism 8.

For co-localization analyses, ImageJ was used to obtain an overlap image and the JACoP plugin to calculate Mander’s coefficients, M1 (for red channel) and M2 (for green channel), as in Dunn et al. (2011). Briefly, Mander’s coefficients quantify the amount of overlap between two different markers in comparison to the total signal of each of them (M1 and M2), this value being not dependent on the intensity of the signal. Then, biological co-occupancy between the markers was easily quantified (e.g., M1 value of 0.3 represents 30% of the marker in the red channel which is also occupied by the marker in the green channel). As a control of random co-localization, we performed a displacement of five pixels in the *X*-axis of the green channel, in order to show the decrease of the overlap.

For the MCC maturation stage, we directly counted cells with aggregates and cells with dispersed distribution of Centriolin at the apical membrane.

### Immunohistochemistry (Ihc)

Mouse lungs were fixed with PFA 4% in phosphate-buffered solution (PBS) (0.1 M, pH 7.4) overnight at 4°C. Samples were rinsed in PBS and then cryoprotected with PBS-sucrose (10%) overnight at 4°C, soaked in embedding medium, frozen onto sectioning blocks, and stored at −80°C. Cryostat sections of 20-μm sections were cut in a longitudinal plan. Sections were thaw-mounted on Superfrost^®^ Plus slides (Menzel-Gläser, Germany) and stored at −20°C. Samples were washed in PBS-Triton X-100 (Sigma, #T8787) (PBS-T) for 15 min. Then, 3% hydrogen peroxide diluted in PBS was added for 45 min. Samples were washed two times in PBS-gelatin (2 g/l) (PanReac, 142060)-Triton X-100 0.25% (PBS-G-T) for 10 min and incubated in PBS-G-T-lysine 1 M (Merck, #62-8365-54) (PBS-G-T-L) for 1 h at RT. Sections were incubated with Jam3 primary antibody (1:150) diluted in PBS-G-T-L in a wet chamber overnight at RT. After that, samples were washed twice in PBS-T for 10 min and once in PBS-G-T for 10 min and then incubated with anti-goat biotinylated IgG secondary antibody (Sigma, #B7014, 1: 200) diluted in PBS-G-T-L in a wet chamber for 2 h at RT. Samples were rinsed twice in PBS-T for 10 min and once in PBS-G-T for 10 min and incubated with streptavidin-HRP (Cell Signaling, #3999S, 1: 100) diluted in PBS-G-T for 2 h at room temperature. Sections were washed twice in PBS-T for 10 min and revealed by using 0.03% 3,3-diaminobenzidine tetrahydrochloride (DAB, Sigma, #868272-85-9) diluted in PBS with 0.025% hydrogen peroxide, creating a brown precipitate where Jam3 was expressed. After reaching appropriate color intensity, the reaction was stopped immersing the slides in PBS-T. Finally, samples were stained with eosin for 10 s and dehydrated in increasing ethanol solutions, washed in Xylol, and mounted in Eukitt. Images were obtained using a Nikon Eclipse 80i microscope and processed using Adobe Photoshop CC 2019.

### SEM

Mouse tracheal epithelial cell cultures differentiated until ALI 14 were fixed in 2.5% glutaraldehyde for 90 min at 4°C, washed in cacodylate 0.2 M, and stained with 1% osmium tetroxide (Sigma) in cacodylate 0.2 M for 2 h at 4°C. Samples were dried by liquid carbon dioxide critical point, gold sputter coated, and visualized in a Quanta 3D FEG (ESEM-FIB; FEI Company, Hillsboro, OR, United States).

### Cilia Beating Assay

Cilia beating frequency was measured in MTECs ALI14. The upper chamber of Transwells was washed with Ca/Mg-PBS and incubated with 5 μl of Dynabeads Streptavidin C1 (Invitrogen) in 300 μl of Ca/Mg-PBS for 10 min at 37°C. After that, the volume of the upper chamber was removed and Transwells were incubated for 2 h at 37°C. Before the recording of the films, 100 μl of Ca/Mg-PBS at 37°C was added in the upper chamber of Transwells. Cilia beating films were obtained using a Motic AE20 microscope, and a standard iPhone 8 was used to record 120-fps movies of around 20 s. A MATLAB app designed *ad hoc* was used to remove the movie background and recognize beating beads as ROIs in the movie. Their trajectories were extracted for all the frames and analyzed in both *x*- and *y*-axis. Finally, a fast Fourier transform function was applied to calculate the beating frequency in both axes. The GUI is available on our web page^1^.

### Basal Body Distribution Pattern Analyses

Basal body staining was used to detect basal bodies in MCCs of MTECs ALI14. Confocal microscopy images were used to distinguish ROIs (Basal Bodies) in a MATLAB custom-made application; interactive drawing allowed MCC selection, and then the average eccentricity and major/minor ratio were extracted for individual cells. Eccentricity was calculated as the distance between the focal points of the minimal ellipse containing the ROI divided by the size of the major axis of this ellipse. In the case of the major/minor axis ratio (Mm ratio), the size of the major axis of the previous ellipse was divided by the size of the minor axis of the same ellipse. Both measures quantify the elongation of the ROI, obtaining longer values in the case of aligned basal bodies.

### Biotinylation Assay

To evaluate the monolayer integrity of MTECs ALI14, Transwells were put on ice and their upper chamber was washed twice in cold Ca/Mg-PBS and incubated with 0.5 mg/ml sulfo-NHS-LC-Biotin (Thermo Scientific) twice for 20 min at 4°C. Then, MTECs were fixed in 4% PFA for 30 min, washed three times in PBS, and stained with Alexa-Fluor 555 streptavidin (Invitrogen, #S21381, 1:500), DAPI at 0.5 μg/ml, and Alexa-Fluor 488 phalloidin (Invitrogen, #A12379, 1:500). Samples were washed three times in PBS and mounted in Vectashield. Finally, images were obtained using an Olympus FV 1000 confocal microscope and were processed using ImageJ and Adobe Photoshop CC 2019.

### TEER Measurements

Tight-junction permeability along the differentiation process of MTECs was tested by measuring TEER every 2 days until ALI14. First of all, the upper chamber of Transwells was washed once in PBS. Then, DMEM at 37°C was added in the upper and lower chambers of Transwells and TEER was measured with Evom3, positioning the electrode in the upper chamber. TEER was measured in kilohms (kΩ). For each time point, a fold change relative to control cells was calculated. Finally, graphs were obtained using Microsoft Excel and GraphPad Prism 8.

### Single-Cell Data Analyses

Single-cell RNA-seq from mouse airways was retrieved from GSE102580, specifically gene expression data for cells determined as multiciliated (Plasschaert et al., 2018). Genes for deuterosomal cells and mature ciliated cells were obtained from bibliography (Bukowy-Bieryłło, 2021), and their single-cell expression (as well as FoxJ1) was obtained in both Jam3-positive (with at least one Jam3 read) and Jam3-negative cells. All these calculations were made in MATLAB.

### Statistical Analyses

Data were analyzed using two-tailed *t*-tests to compare control conditions with different experimental groups (GraphPad Prism). qPCR experiments were performed with at least four biological replicates, and each qPCR reaction was made with two to three technical replicates. Relative expression was calculated using EIF1a as a housekeeping gene.

## Data Availability Statement

The raw data supporting the conclusions of this article will be made available by the authors, without undue reservation.

## Ethics Statement

The animal study was reviewed and approved by the Bioethics Committee for Animal Experimentation of the University of Extremadura (Registry July 7th, 2017).

## Author Contributions

SG-J, JB-L, SD-C, GÁ-H, and CM-Q performed all of the experiments. AR designed and performed the data analysis. FC and JF-M designed the experiments. JC-G designed the experiments, analyzed the data, and wrote the manuscript. All authors contributed to the article and approved the submitted version.

## Conflict of Interest

The authors declare that the research was conducted in the absence of any commercial or financial relationships that could be construed as a potential conflict of interest.

## Publisher’s Note

All claims expressed in this article are solely those of the authors and do not necessarily represent those of their affiliated organizations, or those of the publisher, the editors and the reviewers. Any product that may be evaluated in this article, or claim that may be made by its manufacturer, is not guaranteed or endorsed by the publisher.

## Footnotes

1
http://cellpolaritylab.blogspot.com

## References

[B1] Adler P. N. Wallingford J. B. (2017). From planar cell polarity to ciliogenesis and back: the curious tale of the PPE and CPLANE proteins. *Trends Cell Biol.* 27 379–390. 10.1016/j.tcb.2016.12.001 28153580 PMC5403552

[B2] Ahdieh M. Vandenbos T. Youakim A. (2001). Lung epithelial barrier function and wound healing are decreased by IL-4 and IL-13 and enhanced by IFN-γ. *Am. J. Physiol. Cell Physiol.* 281 C2029–C2038. 10.1152/ajpcell.2001.281.6.c2029 11698262

[B3] Bazzoni G. (2003). The JAM family of junctional adhesion molecules. *Curr. Opin. Cell Biol.* 15 525–530. 10.1016/S0955-0674(03)00104-2 14519386

[B4] Boutin C. Labedan P. Dimidschstein J. Richard F. Cremer H. André P. (2014). A dual role for planar cell polarity genes in ciliated cells. *Proc. Natl. Acad. Sci. U.S.A.* 111 E3129–E3138. 10.1073/pnas.1404988111 25024228 PMC4121795

[B5] Bukowy-Bieryłło Z. (2021). Long-term differentiating primary human airway epithelial cell cultures: how far are we? *Cell Commun. Signal.* 19:63. 10.1186/s12964-021-00740-z 34044844 PMC8159066

[B6] Bustamante-Marin X. M. Ostrowski L. E. (2017). Cilia and mucociliary clearance. *Cold Spring Harb. Perspect. Biol.* 9:a028241. 10.1101/cshperspect.a028241 27864314 PMC5378048

[B7] Cardoso W. V. (2001). Molecular regulation of lung development. *Annu. Rev. Physiol.* 63 471–494. 10.1146/annurev.physiol.63.1.471 11181964

[B8] Cartier-Michaud A. Bailly A. L. Betzi S. Shi X. Lissitzky J. C. Zarubica A. (2017). Genetic, structural, and chemical insights into the dual function of GRASP55 in germ cell Golgi remodeling and JAM-C polarized localization during spermatogenesis. *PLoS Genet.* 13:e1006803. 10.1371/journal.pgen.1006803 28617811 PMC5472279

[B9] Carvajal-Gonzalez J. M. Gravotta D. Mattera R. Diaz F. Bay A. P. Roman A. C. (2012). Basolateral sorting of the coxsackie and adenovirus receptor through interaction of a canonical YXXΦ motif with the clathrin adaptors AP-1A and AP-1B. *Proc. Natl. Acad. Sci. U.S.A.* 109 3820–3825. 10.1073/pnas.1117949109 22343291 PMC3309744

[B10] Carvajal-Gonzalez J. M. Mulero-Navarro S. Mlodzik M. (2016a). Centriole positioning in epithelial cells and its intimate relationship with planar cell polarity. *BioEssays* 38 1234–1245. 10.1002/bies.201600154 27774671 PMC5206807

[B11] Carvajal-Gonzalez J. M. Roman A. C. Mlodzik M. (2016b). Positioning of centrioles is a conserved readout of Frizzled planar cell polarity signalling. *Nat. Commun.* 7:11135. 10.1038/ncomms11135 27021213 PMC4820615

[B12] Chavakis T. Keiper T. Matz-Westphal R. Hersemeyer K. Sachs U. J. Nawroth P. P. (2004). The junctional adhesion molecule-C promotes neutrophil transendothelial migration in vitro and in vivo. *J. Biol. Chem.* 279 55602–55608. 10.1074/jbc.M404676200 15485832

[B13] Coyne C. B. Vanhook M. K. Gambling T. M. Carson J. L. Boucher R. C. Johnson L. G. (2002). Regulation of airway tight junctions by proinflammatory cytokines. *Mol. Biol. Cell* 13 3218–3234. 10.1091/mbc.E02-03-0134 12221127 PMC124154

[B14] Cunningham S. A. Arrate M. P. Rodriguez J. M. Bjercke R. J. Vanderslice P. Morris A. P. (2000). A novel protein with homology to the junctional adhesion molecule Characterization of leukocyte interactions. *J. Biol. Chem.* 275 34750–34756. 10.1074/jbc.M002718200 10945976

[B15] Diaz F. Gravotta D. Deora A. Schreiner R. Schoggins J. Falck-Pedersen E. (2009). Clathrin adaptor AP1B controls adenovirus infectivity of epithelial cells. *Proc. Natl. Acad. Sci. U.S.A.* 106 11143–11148. 10.1073/pnas.0811227106 19549835 PMC2708682

[B16] Dunn K. W. Kamocka M. M. McDonald J. H. (2011). A practical guide to evaluating colocalization in biological microscopy. *Am. J. Physiol. Cell Physiol.* 300 C723–C742. 10.1152/ajpcell.00462.2010 21209361 PMC3074624

[B17] Ebnet K. (2017). Junctional adhesion molecules (JAMs): Cell adhesion receptors with pleiotropic functions in cell physiology and development. *Physiol. Rev.* 97 1529–1554. 10.1152/physrev.00004.2017 28931565

[B18] Fernandez-Borja M. Janssen L. Verwoerd D. Hordijk P. Neefjes J. (2005). RhoB regulates endosome transport by promoting actin assembly on endosomal membranes through Dia1. *J. Cell Sci.* 118 2661–2670. 10.1242/jcs.02384 15944396

[B19] Fielding C. A. McLoughlin R. M. McLeod L. Colmont C. S. Najdovska M. Grail D. (2008). IL-6 Regulates Neutrophil Trafficking during Acute Inflammation via STAT3. *J. Immunol.* 181 2189–2195. 10.4049/jimmunol.181.3.2189 18641358

[B20] Garrido-Jimenez S. Roman A.-C. Carvajal-Gonzalez J. M. (2019). Diminished expression of fat and Dachsous PCP proteins impaired centriole planar polarization in *Drosophila*. *Front. Genet.* 10:328. 10.3389/fgene.2019.00328 31031805 PMC6473044

[B21] Gliki G. Ebnet K. Aurrand-Lions M. Imhof B. A. Adams R. H. (2004). Spermatid differentiation requires the assembly of a cell polarity complex downstream of junctional adhesion molecule-C. *Nature* 431 320–324. 10.1038/nature02877 15372036

[B22] Gravotta D. Carvajal-Gonzalez J. M. Mattera R. Deborde S. Banfelder J. R. Bonifacino J. S. (2012). The clathrin adaptor AP-1A mediates basolateral polarity. *Dev. Cell* 22 811–823. 10.1016/j.devcel.2012.02.004 22516199 PMC3690600

[B23] Günzel D. Yu A. S. L. (2013). Claudins and the modulation of tight junction permeability. *Physiol. Rev.* 93 525–569. 10.1152/physrev.00019.2012 23589827 PMC3768107

[B24] Hartmann C. Schwietzer Y. A. Otani T. Furuse M. Ebnet K. (2020). Physiological functions of junctional adhesion molecules (JAMs) in tight junctions. *Biochim. Biophys. Acta Biomembr.* 1862:183299. 10.1016/j.bbamem.2020.183299 32247783

[B25] Herawati E. Taniguchi D. Kanoh H. Tateishi K. Ishihara S. Tsukita S. (2016). Multiciliated cell basal bodies align in stereotypical patterns coordinated by the apical cytoskeleton. *J. Cell Biol.* 214 571–586. 10.1083/jcb.201601023 27573463 PMC5004441

[B26] Hirano Y. Ode Y. Ochani M. Wang P. Aziz M. (2018). Targeting junctional adhesion molecule-C ameliorates sepsis-induced acute lung injury by decreasing CXCR4+ aged neutrophils. *J. Leukocyte Biol.* 104 1159–1171. 10.1002/JLB.3A0218-050R 30088666 PMC6258282

[B27] Imhof B. A. Zimmerli C. Gliki G. Ducrest-Gay D. Juillard P. Hammel P. (2007). Pulmonary dysfunction and impaired granulocyte homeostasis result in poor survival of Jam-C-deficient mice. *J. Pathol.* 212 198–208. 10.1002/path.2163 17455169

[B28] Kostelnik K. B. Barker A. Schultz C. Mitchell T. P. Rajeeve V. White I. J. (2019). Dynamic trafficking and turnover of JAM-C is essential for endothelial cell migration. *PLoS Biol.* 17:e3000554. 10.1371/journal.pbio.3000554 31790392 PMC6907879

[B29] Kummer D. Ebnet K. (2018). Junctional Adhesion Molecules (JAMs): The JAM-Integrin Connection. *Cells* 7:25. 10.3390/cells7040025 29587442 PMC5946102

[B30] Lee C. Cox R. M. Papoulas O. Horani A. Drew K. Devitt C. C. (2020). Functional partitioning of a liquid-like organelle during assembly of axonemal dyneins. *eLife* 9 1–23. 10.7554/ELIFE.58662 33263282 PMC7785291

[B31] McLoughlin R. M. Witowski J. Robson R. L. Wilkinson T. S. Hurst S. M. Williams A. S. (2003). Interplay between IFN-γ and IL-6 signaling governs neutrophil trafficking and apoptosis during acute inflammation. *J. Clin. Invest.* 112 598–607. 10.1172/JCI17129 12925700 PMC171385

[B32] Nehru V. Voytyuk O. Lennartsson J. Aspenström P. (2013). Rhod binds the rab5 effector rabankyrin-5 and has a role in trafficking of the platelet-derived growth factor receptor. *Traffic* 14 1242–1254. 10.1111/tra.12121 24102721

[B33] Orlova V. V. Economopoulou M. Lupu F. Santoso S. Chavakis T. (2006). Junctional adhesion molecule-C regulates vascular endothelial permeability by modulating VE-cadherin-mediated cell-cell contacts. *J. Exp. Med.* 203 2703–2714. 10.1084/jem.20051730 17116731 PMC2118160

[B34] Pardo-Saganta A. Tata P. R. Law B. M. Saez B. Chow R. D. W. Prabhu M. (2015). Parent stem cells can serve as niches for their daughter cells. *Nature* 523 597–601. 10.1038/nature14553 26147083 PMC4521991

[B35] Pellegrini M. Claps G. Orlova V. V. Barrios F. Dolci S. Geremia R. (2011). Targeted JAM-C deletion in germ cells by Spo11-controlled Cre recombinase. *J. Cell Sci.* 124 91–99. 10.1242/jcs.072959 21147852 PMC3001409

[B36] Plasschaert L. W. Žilionis R. Choo-Wing R. Savova V. Knehr J. Roma G. (2018). A single-cell atlas of the airway epithelium reveals the CFTR-rich pulmonary ionocyte. *Nature* 560 377–381. 10.1038/s41586-018-0394-6 30069046 PMC6108322

[B37] Rawlins E. L. Hogan B. L. M. (2006). Epithelial stem cells of the lung: privileged few or opportunities for many? *Development* 133 2455–2465. 10.1242/dev.02407 16735479

[B38] Rawlins E. L. Okubo T. Xue Y. Brass D. M. Auten R. L. Hasegawa H. (2009). The role of scgb1a1+ clara cells in the long-term maintenance and repair of lung airway, but not alveolar. *Epithelium. Cell Stem Cell* 4 525–534. 10.1016/j.stem.2009.04.002 19497281 PMC2730729

[B39] Rock J. R. Onaitis M. W. Rawlins E. L. Lu Y. Clark C. P. Xue Y. (2009). Basal cells as stem cells of the mouse trachea and human airway epithelium. *Proc. Natl. Acad. Sci. U.S.A.* 106 12771–12775. 10.1073/pnas.0906850106 19625615 PMC2714281

[B40] Roman A. C. Garrido-Jimenez S. Diaz-Chamorro S. Centeno F. Carvajal-Gonzalez J. M. (2019). Centriole positioning: not just a little dot in the cell. *Results Probl. Cell Differ.* 67 201–221. 10.1007/978-3-030-23173-6_8 31435796

[B41] Schmidt H. Braubach P. Schilpp C. Lochbaum R. Neuland K. Thompson K. (2019). IL-13 impairs tight junctions in airway epithelia. *Int. J. Mol. Sci.* 20:3222. 10.3390/ijms20133222 31262043 PMC6651493

[B42] Schreiner R. Frindt G. Diaz F. Carvajal-Gonzalez J. M. Perez Bay A. E. Palmer L. G. (2010). The absence of a clathrin adapter confers unique polarity essential to proximal tubule function. *Kidney Int.* 78 382–388. 10.1038/ki.2010.166 20531453 PMC3684398

[B43] Sironen A. Shoemark A. Patel M. Loebinger M. R. Mitchison H. M. (2020). Sperm defects in primary ciliary dyskinesia and related causes of male infertility. *Cell. Mol. Life Sci.* 77 2029–2048. 10.1007/s00018-019-03389-7 31781811 PMC7256033

[B44] Tadokoro T. Wang Y. Barak L. S. Bai Y. Randell S. H. Hogan B. L. M. (2014). IL-6/STAT3 promotes regeneration of airway ciliated cells from basal stem cells. *Proc. Natl. Acad. Sci. U.S.A.* 111 E3641–E3649. 10.1073/pnas.1409781111 25136113 PMC4156689

[B45] Tissir F. Qu Y. Montcouquiol M. Zhou L. Komatsu K. Shi D. (2010). Lack of cadherins Celsr2 and Celsr3 impairs ependymal ciliogenesis, leading to fatal hydrocephalus. *Nat. Neurosci.* 13 700–707. 10.1038/nn.2555 20473291

[B46] Usami F. M. Arata M. Shi D. Oka S. Higuchi Y. Tissir F. (2021). Intercellular and intracellular cilia orientation is coordinated by CELSR1 and CAMSAP3 in oviduct multi-ciliated cells. *J. Cell Sci.* 134:jcs257006. 10.1242/jcs.257006 33468623

[B47] Vladar E. K. Bayly R. D. Sangoram A. M. Scott M. P. Axelrod J. D. (2012). Microtubules enable the planar cell polarity of airway cilia. *Curr. Biol.* 22 2203–2212. 10.1016/j.cub.2012.09.046 23122850 PMC3518597

[B48] Vladar E. K. Nayak J. V. Milla C. E. Axelrod J. D. (2016). Airway epithelial homeostasis and planar cell polarity signaling depend on multiciliated cell differentiation. *JCI Insight* 1:e88027. 10.1172/jci.insight.88027 27570836 PMC4996276

[B49] Woodfin A. Voisin M. B. Beyrau M. Colom B. Caille D. Diapouli F. M. (2011). The junctional adhesion molecule JAM-C regulates polarized transendothelial migration of neutrophils in vivo. *Nat. Immunol.* 12 761–769. 10.1038/ni.2062 21706006 PMC3145149

[B50] Wyss L. Schäfer J. Liebner S. Mittelbronn M. Deutsch U. Enzmann G. (2012). Junctional Adhesion Molecule (JAM)-C Deficient C57BL/6 Mice develop a severe hydrocephalus. *PLoS One* 7:e45619. 10.1371/journal.pone.0045619 23029139 PMC3445510

[B51] Yang Y. Riccio P. Schotsaert M. Mori M. Lu J. Lee D. K. (2018). Spatial-temporal lineage restrictions of embryonic p63+ progenitors establish distinct stem cell pools in adult airways. *Dev. Cell* 44 752–761.e4. 10.1016/j.devcel.2018.03.001 29587145 PMC5875454

[B52] Zen K. Babbin B. A. Liu Y. Whelan J. B. Nusrat A. Parkos C. A. (2004). JAM-C is a component of desmosomes and a ligand for CD11b/CD18-mediated neutrophil transepithelial migration. *Mol. Biol. Cell* 15 3926–3937. 10.1091/mbc.E04-04-0317 15194813 PMC491847

