# Supplementary material for: Junctional Adhesion Molecule 3 Expression in the Mouse Airway Epithelium Is Linked to Multiciliated Cells
Source: Front Cell Dev Biol. 2021 Jul 28;9:622515. doi: 10.3389/fcell.2021.622515 (PMC8355548; doi:10.3389/fcell.2021.622515)
Supplement: Supplementary file 1 [file Data_Sheet_1.PDF]

Supplementary files

Supplementary table I. Oligonucleotides used to make Jam3 shRNAs, Jam2 shRNAs and oligonucleotides to analyze tight junction and cell lineage markers expression by qPCR.

**RNA**	**Sequence**
shJam3 1 F	CCGGCTTATTGTTCTTGCTGTGATTCTCGAGAATCACAGCAAGAACAATAAGTTTTTG
shJam3 1 R	AATTCAAAAACTTATTGTTCTTGCTGTGATTCTCGAGAATCACAGCAAGAACAATAAG
shJam3 2 F	CCGGGCCAAACCACATATGTGTATTCTCGAGAATACACATATGTGGTTTGGCTTTTTG
shJam3 2 R	AATTCAAAAAGCCAAACCACATATGTGTATTCTCGAGAATACACATATGTGGTTTGGC
shJam2 1 F	CCGGCGTCAAGAAGTCACAGTAATACTCGAGTATTACTGTGACTTCTTGACGTTTTTG
shJam2 1 R	AATTCAAAAACGTCAAGAAGTCACAGTAATACTCGAGTATTACTGTGACTTCTTGACG
shJam2 2 F	CCGGGCAATTCAACATGATTTCCAACTCGAGTTGGAAATCATGTTGAATTGCTTTTTG-
shJam2 2 R	AATTCAAAAAGCAATTCAACATGATTTCCAACTCGAGTTGGAAATCATGTTGAATTGC
mJam3 F	CCACAACCATGGCGCTGAGCCGG
mJam3 R	CCATGGTTGTGGTCCAGATAACAAAGGACG
Foxj1 F	GTTTCATCCACCCATGTTCC
Foxj1 R	GTAGGACCCTTCTGGGCTTC
Scgb1a1 F	CATCATGAAGCTCACGGAGA
Scgb1a1 R	AGGTGAGATGCTCGCAGTTT
Krt5 F	GGAGCTGGTCTCAAAGATG
Krt5 R	TCCAGCAGCTTCCTGTAGG
Jam1 F	TCAGTGTCCCCTCCTCTGTC
Jam1 R	GCATCTGCTGTAAGCATGGA
Jam2 F	TGACTGGAAGTGTGGTGGAG
Jam2 R	CCTTTTGGATTCCCTAGCAA
Jam3 F	TACAGCTGGTACCGCAATGA
Jam3 R	GAGTGCCTGTCTCCGAGTTC
Eif1a F	AATGTGCTTTGACGGTGTGA
Eif1a R	TGATTTTGGCATGTTCTGGA

Supplementary Figure 1

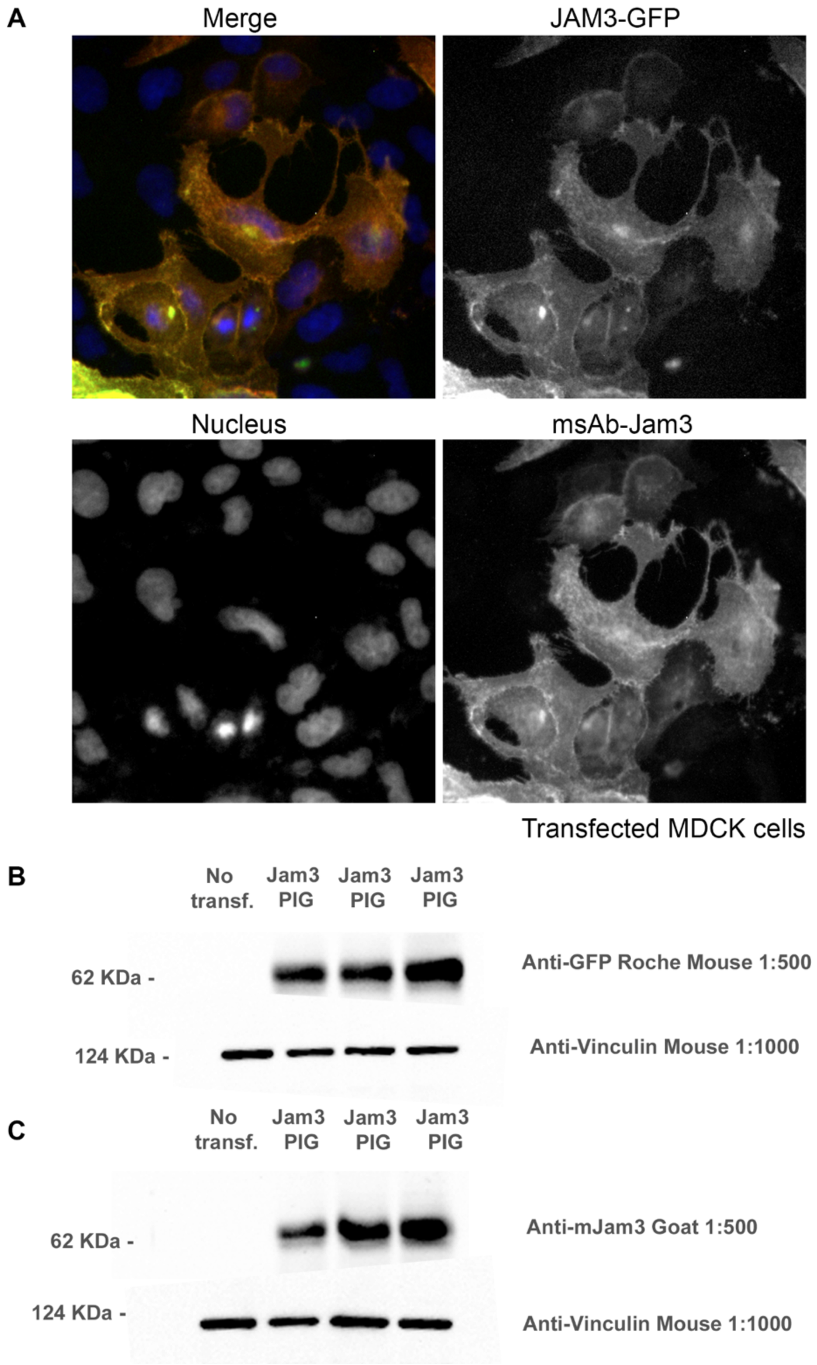

Supplementary Figure 1: Jam3 AF1213 antibody validation.
(A) Jam3 immunofluorescence in MDCK transfected with mouse Jam3-puromycin-IRES-GFP showing the coincidence of anti-Jam3 staining and Jam3-GFP signal. **(B-C)** Western-blots images for anti-GFP, anti-Jam3, and anti-vinculin in control (mCherry transfected HEK293 cells) or Jam3-PIG transfected 293T to test ability of anti-Jam3 to recognize Jam3 in WB.

Supplementary figure 2

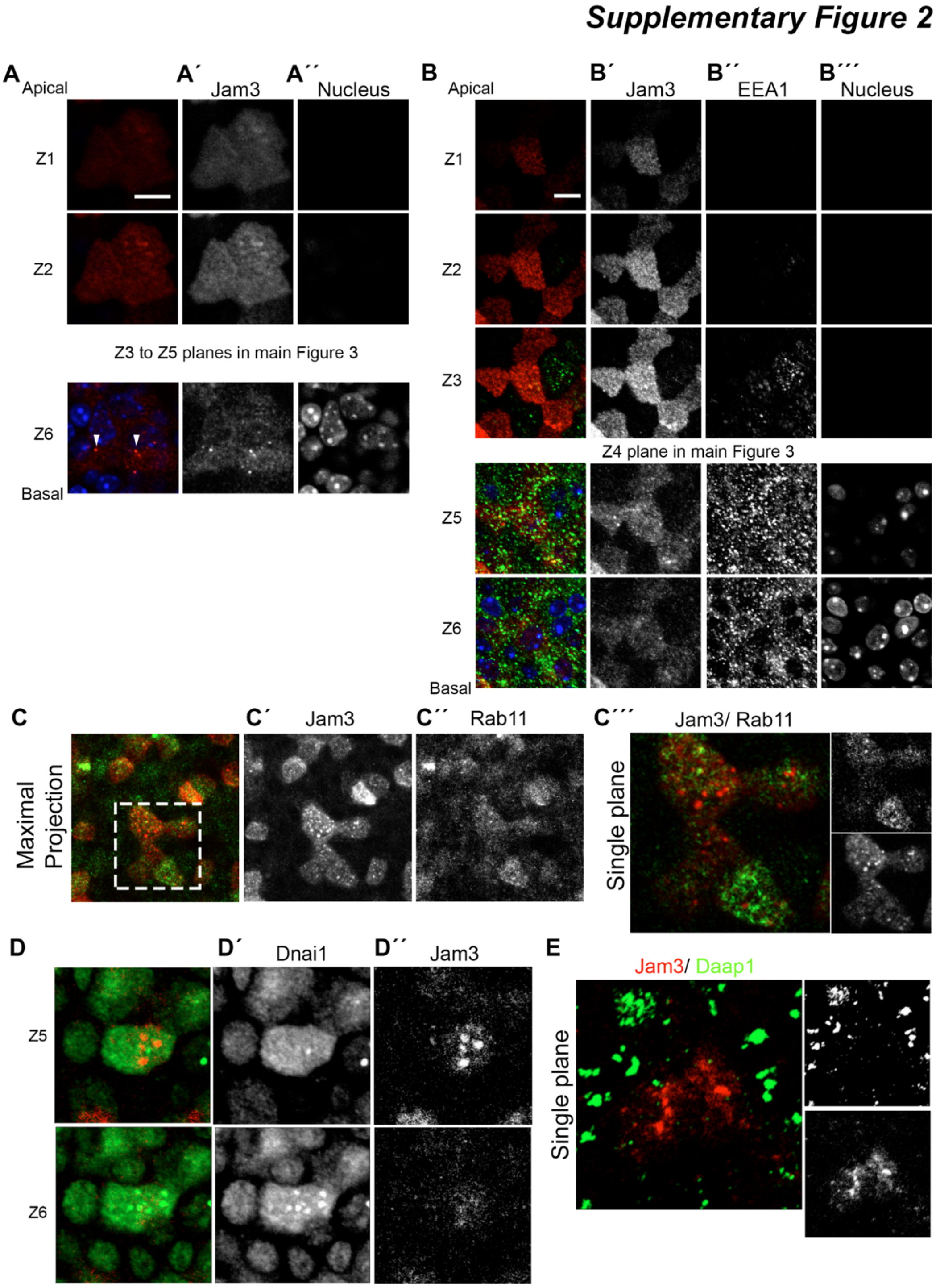

Supplementary Figure 2: Jam3 does not co-localize with Rab11 positive endosomes. **(A)** Serial confocal Z-planes from apical to more basal ones (Z1, Z2 and Z6) of Jam3 in MCCs. White arrows point Jam3 localization at apically located endosomes. **(B)** Serial confocal Z-planes (Z1 to Z6, except Z4 present in main figure 3) of Jam3 colocalization with EEA1 in MCCs. (**C**) Immunofluorescence of Jam3 (red) staining in combination with Rab11 (green) in MTECs at ALI14. (**D**) Immunofluorescence of Jam3 (red) staining in combination with Dnai1 (green) in MTECs at ALI14**.** (**E**) Immunofluorescence of Jam3 (red) staining in combination with Daap1 (green) in MTECs at ALI14**.**

Supplementary Figure 3

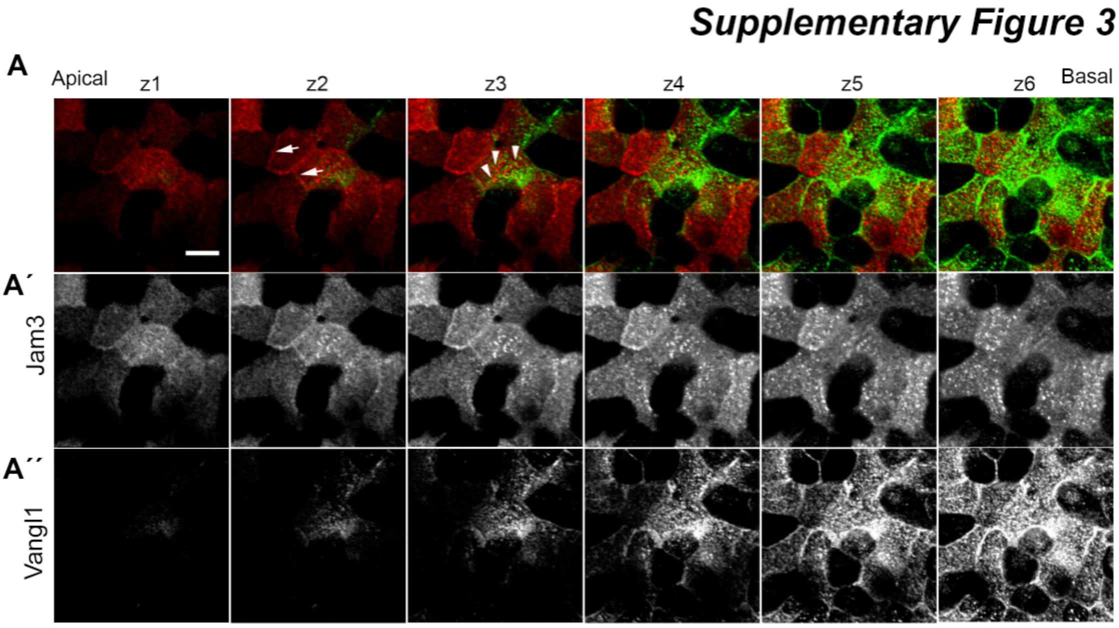

Supplementary Figure 3: Jam3 and Vangl1 colocalization analyses.
(**A**) Serial confocal Z-planes (Z1 to Z6) of Jam3 in red (grey in A´) colocalization with Vangl1 in green (grey in A´´) in MCCs. White arrows point Jam3 localization at cell-cell contacts which are negative for Vangl1. White arrow heads denoted Jam3 endosomes which are Vangl1 negative. The step size between Z plane is 1µm. Scale bar in A represents 10µm.

Supplementary Figure 4

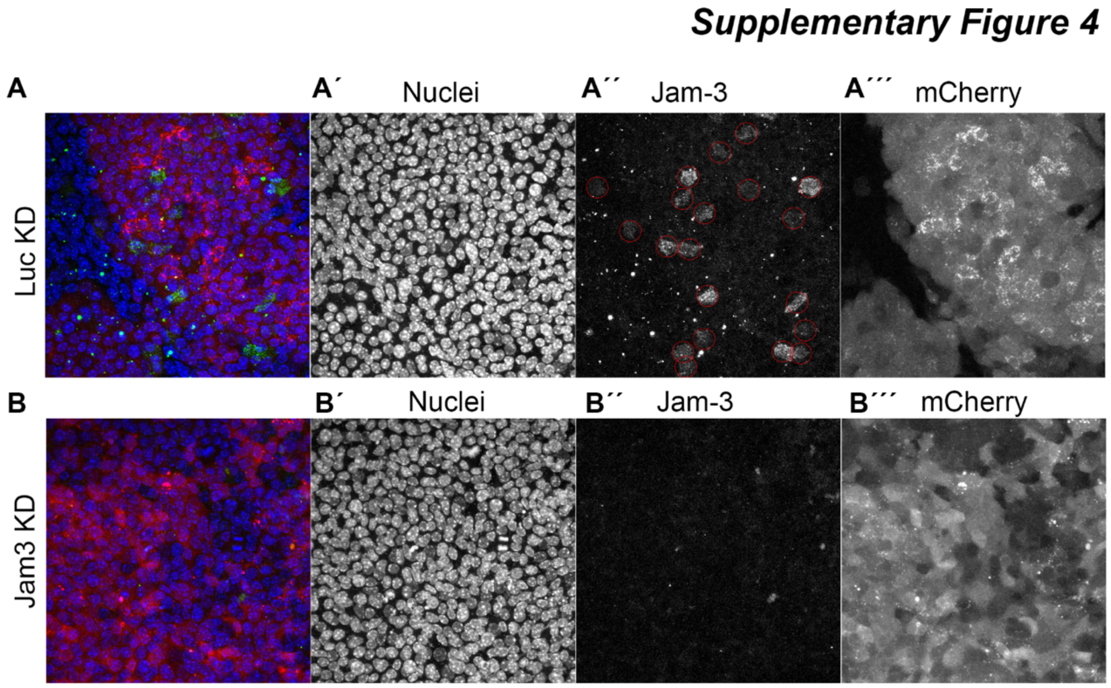

Supplementary Figure 4: Jam3 knockdown in differentiated MTECs. Immunofluorescence images of anti-Jam3 (green) in infected MTECs (mcherry, red) with shRNA against Luciferase (shLuciferase) (A) or Jam3 (sh1 and sh2 Jam3)(B) to test Jam3 knockdown in MTECs at ALI14. (A´) Jam3 positive cells circled in red.

Supplementary figure 5

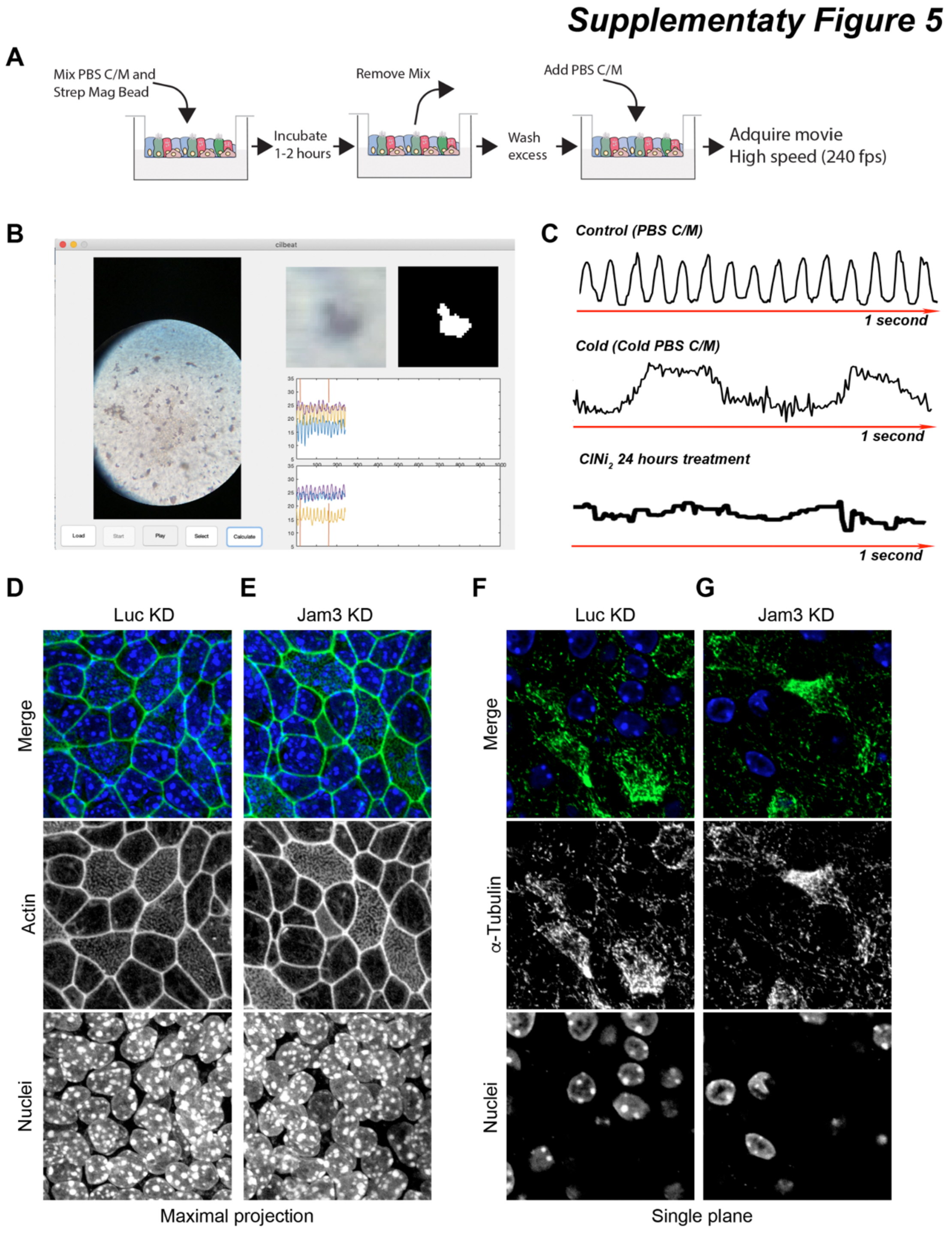

Supplementary Figure 5: A new method to measure cilia beating in MTECs in culture.

(A) Schematic representation of the assay developed to measure cilia beating described in Materials and Methods. **(B)** Snapshot of the GUI developed to measure cilia beating. **(C)** Representation of cilia beating waves in one second in different experimental conditions. (**D-E**) Actin staining using phalloidin in control (Luc KD) and Jam3 KD cells. (**F-G**) α-tubulin staining in control (Luc KD) and Jam3 KD cells.

Supplementary figure 6

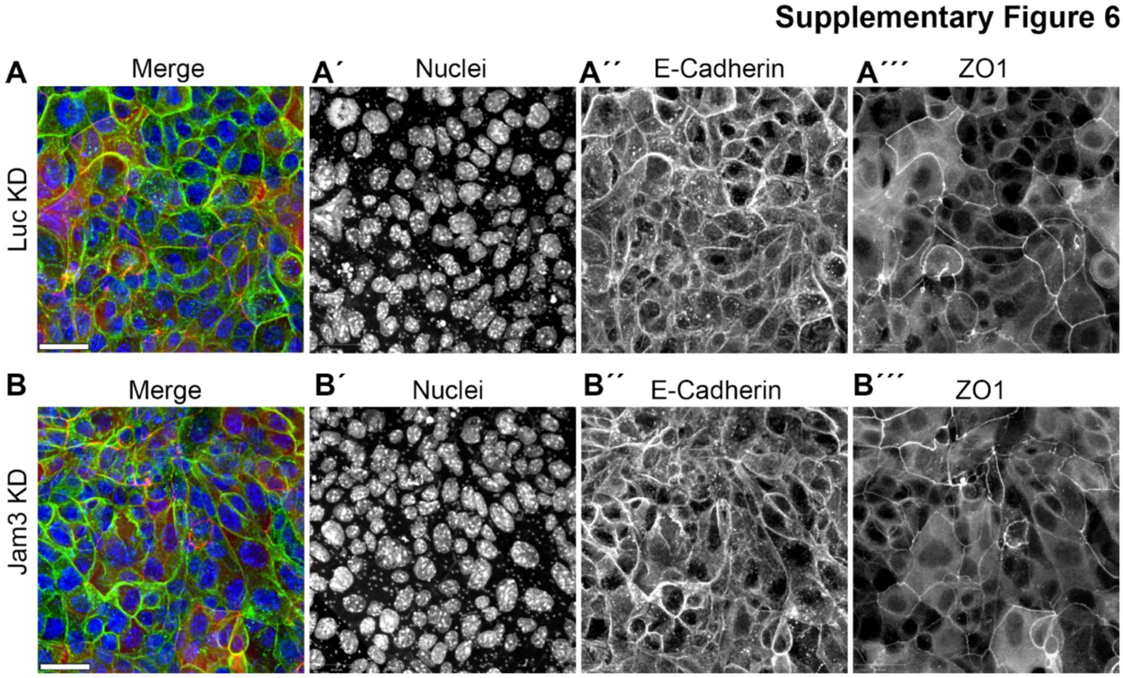

Supplementary Figure 6: Cell-cell adhesion molecules localization in control and Jam3 KD cells.

(A) Maximal projection of confocal image at pre-ALI stage for ZO1 in red (grey in **A´´´**), E-cadherin in green (grey in **A´´**) and nucleus in blue (**A´**) in control cells. **(B)** Maximal projection of confocal image at pre-ALI stage for ZO1 in red (grey in **B´´´**), E-cadherin in green (grey in **B´´**) and nucleus in blue (**B´**) in Jam3 KD cells. Scale bar in A and B represents 30µm.
